# Girls-only vs. mixed-gender groups in the delivery of a universal wellness programme among adolescents: A cluster-randomized controlled trial

**DOI:** 10.1371/journal.pone.0198872

**Published:** 2018-06-18

**Authors:** Reut Agam-Bitton, Wiessam Abu Ahmad, Moria Golan

**Affiliations:** 1 Department of Nutritional Sciences, Tel Hai College, Upper Galilee, Israel; 2 Hebrew University-Hadassah Braun School of Public Health and Community Medicine, Jerusalem, Israel; 3 Shahaf, Community Services for the Management of Weight-Related Problems, Tel Aviv, Israel; TNO, NETHERLANDS

## Abstract

**Background:**

Investigation of the optimal setting for body image prevention programmes is important to maximize the outcomes of such programmes.

**Objectives:**

We examined the preferred setting for a school-based wellness programme called “In Favour of Myself".

**Methods:**

A total of 259 girls (mean 13.82±0.64 years) were divided into a girls-only intervention group, a mixed-gender intervention and a waiting list control group. The participants completed self-report questionnaires at baseline, post-intervention (2 months) and at follow-up (3 months) examining changes in self-esteem, media literacy, body image and risk factors for eating disorders. The intervention group participants also completed a satisfaction questionnaire.

**Results:**

Both intervention groups demonstrated statistically significant improvements in identifying advertising strategies (p<0.01) compared with the controls, with the girls-only arm (p<0.001) showing better results. Compared with the girls-only arm and the control group, the mixed-gender group demonstrated statistically significantly greater improvements in the internalization of pressure for thinness (p<0.004), the body-esteem appearance subscale (p<0.025) and body-esteem body-weight subscale (p<0.012) as well as reductions in their perceived current body silhouettes and in the gap between their current and ideal body image (p<0.003). Body dissatisfaction was increased following the programme, although not in a statistically significant manner, with the worst negative effect observed in the girls-only arm. All other differences among the study arms did not show statistically significant differences.

Mediation models revealed that body-esteem was directly mediated by group, with statistically significant mediation only in the mixed group. Current body image was mediated indirectly by group through media literacy (i.e., recognizing advertisement strategies and internalization of pressure for thinness), with statistical significance only in the mixed-gender arm compared with the girls-only arm.

Higher programme satisfaction was reported in the mixed-gender group (91%) vs. the girls-only groups (79%).

**Conclusions:**

These outcomes provide preliminary evidence indicating the superiority of a mixed-gender setting compared with a girls-only setting for delivering prevention programmes to 13- to 14-year-old adolescents to enhance their media literacy, positive self-esteem and body image.

**Trial registration:**

NCT02653586

## Introduction

Self-esteem and body image worsen during adolescence as a result of the physical, social and cognitive changes that occur during that period [1]. Because self-esteem and body image are both considered to protect against risk behaviours in adolescents [2], it is important to preserve and promote them through universal prevention programmes. Prevention programmes for body dissatisfaction and eating disorders usually tackle media literacy, body functionality, appreciation of a broad array of body shapes and characteristics, development of critical social perspectives regarding the body, and self-esteem enhancement [2]. Many of these programmes only target girls [3].

School-based programs are the preferred setting for most of adolescents’ prevention programs [4]. The school setting offers potential space for ongoing interactions with other adolescents of the same age, through the sharing of experiences, opinions and attitudes [5]. The effectiveness of school-based programmes has been reported to be higher among girls than boys [3]. Thus, it is not surprising that teachers in our country prefer that classes be divided according to gender and that programmes that address body issues be administered to girls-only groups.

The National Ministry of Education in our country currently uses the prevention programme “In Favour of Myself” for delivery in schools. Thus, it is crucial to study the setting issues related to this programme. This programme was previously examined in mixed-gender groups (13–14 years). At a 3-month follow-up session, girls exhibited a significant increase in self-esteem (school achievement, parents' perceptions) and a significant decrease in the contribution of appearance to self-esteem (*p* < 0.05), as well as significant improvement in ideal body image (*p* < 0.05); the boys did not demonstrate these changes [6].

Nevertheless, the question of the most effective setting for girls—a mixed-gender group or a single-gender group—has only recently been addressed [7]. The influence of the “Happy Being Me” programme on body image was assessed among 200 girls (74 in a single-gender group, 73 in a mixed-gender group and 53 in a control group) in 7^th^ grade in Melbourne, Australia. This 6-session programme lasts 50 min per session. At post-intervention, although significant improvements were observed in body dissatisfaction, internalization of the thin ideal, appearance comparisons and self-esteem, the effects were found to be low (0.04 to 0.06), and no differences were found between the single-gender and mixed-gender groups. Another study addressing the issue of setting and gender has been recently published [8]. “The All Body Project 4”, a two-session programme lasting 2 h per session, was delivered to 185 US college students (115 girls and 70 boys) aged 19.9 years. The participants were divided into 3 research arms: female only (n = 38), mixed gender (41 females and 36 males) and a control group (36 females and 34 males). At post-intervention, there was no distinct superiority of the mixed vs. female-only setting, as expressed by the women’s body satisfaction and eating-disorder pathology. In contrast, other studies have shown that eating disorder prevention programmes tend to be more effective when delivered to at-risk females or when solely addressed to either at-risk females or at-risk males [9]. As these studies were conducted with different age groups, the question of the superior setting for adolescent girls remains unanswered.

In the present study, we examined the participants’ satisfaction with the programme and the impact of “In Favour of Myself” among adolescent girls when delivered in girls-only groups compared with mixed-gender groups.

We hypothesized that the girls-only groups would demonstrate superiority compared with the control and mixed-gender groups in most of the studied variables.

## Methods

### Study design and population

A cluster randomized controlled trial was conducted in all of the three post-primary schools located in the north of Israel. The research protocol was approved by the Tel Hai Institutional Review Board on August 2016, prior to enrolment. Informed consent was signed by all participants and their parents in October 2016. The programme delivery started in November 2016 and terminated in January 2017; the follow-up assessment was performed in April 2017. The registered research number is NCT02653586.

A total of 259 adolescent girls in the 7^th^ and 8^th^ grades from three middle schools participated in this study. A research assistant who was blinded to the characteristics of the classes used the randomization function in Microsoft Excel to randomize the classes at each school to the control and intervention groups. The same function was used for the randomized allocation of the intervention groups between the mixed-gender arm and the single-gender arm. The randomization and programme delivery units were the class. After randomization, each school’s contact person was informed of the study arm to which each class was allocated. In each school, one class was allocated to the mixed-gender group, one to the girls-only group and one to the waiting list control group. At the same time, that girls in the groups were in the intervention, the boys participated in a game activity with a gym teacher. Overall, the mixed-gender intervention groups included 84 participants, the girls-only intervention groups included 88 participants, and the control groups included 87 participants. The entire classes that were allocated to the intervention arm, received the programs (intact classes allocation), starting in November 2015. Since these classes are often too large for experiential group program delivery, each class was divided into two equal groups of 15 participants (we had twelve intervention groups and six control groups).

**Fig 1:**
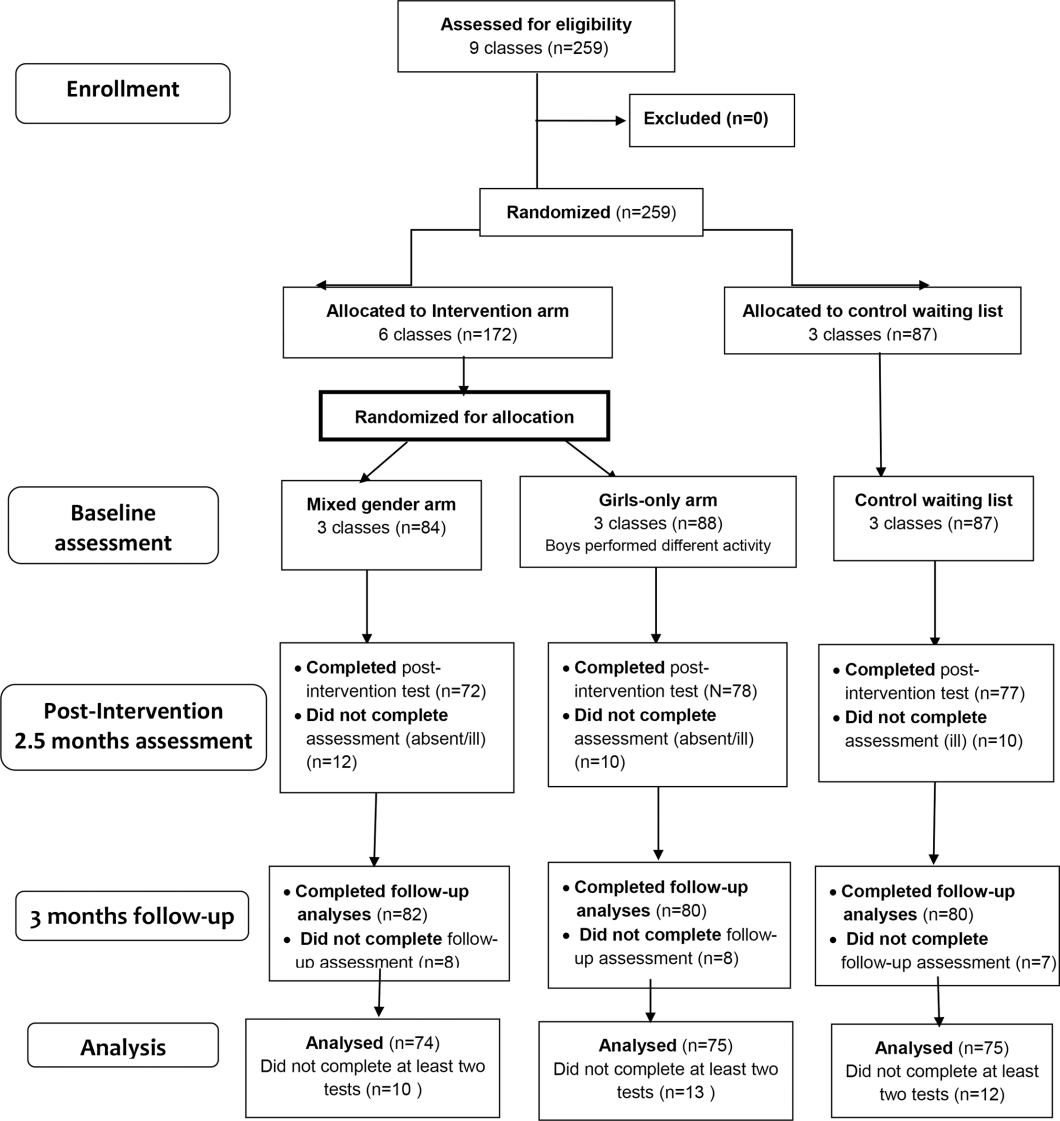
A flow diagram of the participants.

All study participants completed similar computerized questionnaires at baseline, post-intervention (at 2 months) and at a 3-month follow-up session, within the school setting (Fig 1). The research student was in charge of making sure all classes completed this assessment procedure in a similar manner. A blinded statistician performed the data analysis.

### Description of the intervention

“In Favour of Myself” is an interactive wellness programme aimed at promoting positive self-esteem, body image and media literacy among adolescents. The programme was developed by a group of experts and initiated by the Dove Self-Esteem Fund; it was previously assessed among middle adolescents [6,10]. The intervention includes 9 weekly 90-min sessions. The program’s content and topics are described in Table 1. Female undergraduate or graduate students from the department of education or nutrition delivered the programme. Although both genders were invited to participate in this academic course (“Intense supervised delivery of community-based groups”), only females were registered during these two years. All programme facilitators participated in the students’ didactic demonstration and class discussion for each session, and the students received ongoing group and individual supervision while running the groups.

**Table 1:** Session content and descriptions.

Session	Topic	Description
1	Preliminary meeting: introduction and contract establishment.	Introducing the programme and its goals, forming the groups, establishing a group contract.
2	Adolescence and self-esteem	Discussion of the changes that occur in adolescence, interactive activity on the component of self-esteem, its development, and how it is influenced by communication.
3	Advertising–effect of mass media on self-esteem.	Interactive activity and discussion about the impact of media on self-esteem, knowledge of advertising strategies, and development of critical thinking in relation to the media.
4	Stereotypes–the impact of stereotypes on self-esteem.	Interactive activity and discussion about the impact of stereotypes on self-esteem, our values and our position in relation to the individual.
5	Beauty myth	Beauty in history and different cultures and changes in beauty criteria, respecting differences and a variety of sizes, putting the modern beauty myth on trial and questioning the automatic association between beauty and happiness.
6	The power of words, intrapersonal communication, body image and self-esteem.	Sharing personal stories of self-esteem and body image, exercising cognitive dissonance to challenge destructive perceptions and messages.
7	The power of words, interpersonal communication and self-esteem.	Sharing and challenging negative interpersonal communication using role play.
8	The self: external and internal	Creating artistic metaphors for ‘the self’ (internal and external), and sharing commonalities and differences.
9	Final meeting: where do we go next?	Summation of the contents, position future stances.

### Study outcomes

At baseline, the participants completed demographic data form. Thereafter, the participants completed a computerized questionnaire that included the described variables. At post-intervention, participants in the intervention group also completed a satisfaction questionnaire and an attendance report. The following variables were examined in the study:

#### Self-esteem

This variable was assessed using the Rosenberg self-esteem scale (RSES). The scale consists of 10 items scored on 4 points: (1) strongly disagree, (2) disagree, (3) agree, (4) strongly agree. The final score is created by summing the item scores and, thus, ranges from 10–40. A higher score indicates higher self-esteem [11]. Cronbach’s alphas for the three assessments points in the present study at baseline were 0.84, 0.87 & 0.86.

#### Media literacy

We assessed each participant’s ability to recognize the strategies employed by media advertisements (exaggeration, illusion, appeal to emotion, romanticization, idealization and intimidation), while ignoring those that are mentioned in the scale but are not used (authenticity, appeal to rationality and more). The final score is achieved by summing the points. A higher score is indicative of better media literacy. The validation and reliability were tested in previous research [10]. Cronbach’s alphas for the three assessments points in the present study at baseline were 0.94, 0.87 & 0.90.

#### Sociocultural Attitudes Towards Appearance Questionnaire-3 (SATAQ-3)

We used the pressure subscale of this questionnaire (includes 7 items) to further assess media literacy. Items are rated on a 5-point scale: (1) always, (2) often, (3) sometimes, (4) rarely, (5) never. The total score is based on computing the average. A higher score indicates higher pressure from the media to change one's look [12]. Cronbach’s alphas for the three assessments points in the present study at baseline were 0.87, 0.89 & 0.91.

#### Drive for thinness and body dissatisfaction

These factors were assessed using two subscales from the Eating Disorder Inventory-2 (EDI-2): the drive for thinness (7 items) and body dissatisfaction (9 items). The items are rated on a 6-point scale: (1) never, (2) rarely, (3) sometimes, (4) often, (5) usually, and (6) always. The final score is computed by summing the points. Higher scores indicate a drive for thinness and/or higher body dissatisfaction [13]. Cronbach’s alphas at the three assessments points in the present study at baseline were 0.83, 0.84 & 0.87.

Body image was measured using the Figure Rating Scale (FRS), which contains 9 silhouettes of body images. Participants chose the silhouettes that represented their current and ideal body images (how they want to look like) [14]. The gap between the scores of the current and ideal silhouettes were calculated. A larger gap represents higher body dissatisfaction.

#### Body-esteem (BES)

This questionnaire examines self-esteem of body and physical appearance and consists of 3 subscales: appearance (10 items), weight (8 items) and attribution to others (5 items). Items are rated on a 5-point scale: (1) never, (2) rarely, (3) sometimes, (4) often, and (5) always. A higher score indicates higher body-esteem [15]. Cronbach’s alphas at the three assessments points in the present study at baseline were 0.92, 0.89 & 0.90.

The group facilitators monitored the attendance rate at each session.

Satisfaction with the programme was evaluated only at programme termination. The participants were asked to rate 4 statements regarding satisfaction with the programme (level of satisfaction, intention to recommend the programme to friends, most liked session and most disliked session).

### Statistical analysis

#### Sample size

The sample size was calculated using the EpiTools epidemiological calculation, based on data from previous studies: the sds were 5.21 and 5.14, and the mean was approximately 23. The ICC (intraclass correlation) was 0.02 (6), A significant difference was indicated by a change of 2.5 scores in the average score, with a power of 80%, and α = 0.05. The calculation yielded a sample size of approximately 80 in each group. Assuming a 5% expected dropout rate (as school is obligatory), the calculated sample size was 84 participants in each study arm.

#### Date analysis

Statistical analyses were conducted using SPSS software, version 22. To enable the intention to treat analysis, multiple imputation was used to account for the missing data [16]. Chi-square tests and one-way ANOVA were conducted to compare the groups at baseline. Because baseline age and residence type differed significantly between the study arms, data were adjusted for these two variables. Differences between groups and over time were tested using a random effects analysis of covariance, with class as a random factor, group (intervention girls-only, intervention mixed and control) as a fixed factor, and baseline age as a continuous covariate, followed by the Bonferroni test to adjust for multiple comparisons and to control for Type I errors.

Mediation analysis was performed to study the interdependencies between the post-intervention variables and follow-up variables when a statistically significant difference was observed between groups at follow-up. These models were run using the SPSS macro PROCESS [17]. A p-value < 0.05 was considered to be statistically significant. All reported *P*-values are two-tailed.

## Results

### Baseline characteristics of the participants

At baseline, the study groups (girls-only intervention, mixed-gender intervention, and control) differed in age and residence type (Table 2). Thus, data were adjusted for these two variables. In general, the participant ranged in age between 13.3 and 14.3 years, and the recruited population was considered to be lower middle class.

**Table 2:** Personal and sociodemographic characteristics of the studied population.

Variable	Girls-only intervention (n = 88)	Mixed-gender intervention (n = 84)	Control (n = 87)	*p*-value
Age, M (SD)	13.8 (0.6) ^**a**^	13.6(0.6) ^**a**^	14.0(0.7) ^**b**^	<0.001
Number of siblings	3.6 ± 1.8	3.3 ± 0.9	3.4 ± 1.4	0.361
**Family structure (%)**				
Parents married	76.1	85.4	84.1	0.223
Parents divorced or separated	21.6	10.4	10.3	
Single-parent family	2.3	2.1	4.7	
other	-	2.1	0.9	
**Attitude to religion (%)**				
Religious	3.4	6.3	5.6	0.321
Traditional	46.6	58.3	57.9	
Secular	50.0	35.4	36.4	
**Place in birth order**				
Eldest	28.4	20.8	29.9	0.380
Middle child	38.6	47.9	33.6	
Younger	30.7	31.3	30.8	
Single child	2.3	-	5.6	
**Economic situation (%)**				
Rich family	10.2	12.5	22.4	0.053
Living comfortably	83.0	87.5	72.0	
Restricted living	6.8	-	5.6	
**Father’s education (%)**				
Elementary school	5.7	2.1	3.7	0.285
High school	37.5	37.5	50.5	
Professional education	37.5	47.9	26.2	
BA	10.2	6.3	12.1	
MA	9.1	6.3	7.5	
**Mother’s education (%)**				
Elementary school	-	2.1	1.9	0.291
High school	35.2	33.3	40.2	
Professional education	22.7	39.6	29.9	
BA	29.5	16.7	17.8	
MA	12.5	8.3	10.3	
**Residence type (%)**				
City	58.0 ^**a**^	58.3 ^**b**^	86.0 ^**b**^	<0.001
Private farming community—village	21.6 ^**a**^	16.7 ^**b**^	2.8 ^**b**^	
Cooperative village	20.5 ^**a**^	25.0 ^**a**^	11.2 ^**a**^	

^**a,b**^ the mean difference is significant between groups at the p<0.05 level.

At baseline, between the three groups, no significant differences were observed in the outcome variables, except for media literacy, which was higher in the mixed-gender group (*F*_(2, 258)_ = 5.42,*p* = 0.005; partial *η*^2^ = 0.045). Because the effect size of the difference was small, no adjustment was made. When we activated the adjustment, the same results were obtained. The values observed in this study population were similar to those reported in other samples [6].

### Intervention effects

#### Self-esteem

Random-effects ANCOVA revealed no significant difference between the groups over time, in terms of changes in self-esteem (*F*_(2,234)_ = 2.958,*p* = 0.054) (Table 3). Nevertheless, a reduction in self-esteem was noted among the mixed-gender participants at the programme conclusion, with a *p* value that was nearly significant and a small effect size. This reduction was improved at follow-up and reached the highest level of self-esteem at the follow-up among participants in the mixed-gender groups, although it was not statistically significant.

**Table 3:** Change over time in self-esteem (raw means [SD] and adjusted means [95% CI]).

Outcome variables	Intervention girls-only group		Intervention mixed group		Control group		Between-group differences^a^	
	Raw mean (SD)	Change from baseline	Raw mean (SD)	Change from baseline	Raw mean (SD)	Change from baseline	*p*	*ES*
		Adjusted mean (95% CI)		Adjusted mean (95% CI)		Adjusted mean (95% CI)		
**Rosenberg self esteem**								
Baseline	22.23 (5.42)		22.77 (5.14)		23.03 (5.08)			
Post-intervention	21.71 (6.48)	-0.26 (-1.72; 1.20)	21.54 (5.47)	-2.07 (-3.67;-0.47)	23.37 (5.42)	0.24 (-1.13; 1.61)	0.054	0.025
3-month follow-up	22. 74 (5.63)	1.36 (-0.16; 2.88)	23.66 (4.72)	-0.14 (-1.81;1.52)	22.99 (5.30)	0.43 (-1.00; 1.85)	0.292	0.010

^**a**^ p-value and partial-Eta square from random effects analysis of covariance. Partial η2: 0.02 –small, 0.13 –medium, 0.26 –large. Adjusted mean scores are controlled for age and residence type.

#### Media literacy

Recognizing advertisement strategies and internalization of pressure towards appearance were used to assess media literacy (Table 4). In terms of recognizing advertisement strategies, the random-effects ANCOVA found a statistically significant interaction between group and time. Both intervention groups (girls-only and mixed-gender) demonstrated significant improvements in recognizing advertisement strategies at post-intervention and at follow-up, compared with the control group, with a medium effect size (*F*_(2, 234)_ = 25.342, *p* = 0.001; partial *η*^2^ = 0.16).

**Table 4:** Change over time in media literacy (recognizing media strategies and internalization of pressure towards appearance (raw means [SD] and adjusted means [95% CI]).

Outcome variables	Intervention girls group		Intervention mixed group		Control group		Between-group differences^c^	
	Change from baseline		Change from baseline		Change from baseline			
	Raw mean (SD)	Adjusted mean (95% CI)	Raw mean (SD)	Adjusted mean (95% CI)	Raw mean (SD)	Adjusted mean (95% CI)	*p*	*ES*
**Advertisement strategies**								
Baseline	2.45 (1.01)		2.52 (1.53)		2.30 (1.43)			
Post-intervention	3.06 (1.72)^**a**^	1.33 (0.74; 1.93)	3.29 (1.40) ^**ab**^	0.93 (0.27; 1.58)	2.50 (1.38)^**b**^	0.25 (-0.31; 0.81)	**0.001**	0.060
3 months follow-up	3.30 (1.46)^**a**^	1.40 (0.81; 1.99)	3.70 (1.66) ^**a**^	1.19 (0.55; 1.84)	2.46 (1.50)^**b**^	0.22 (-0.33; 0.77)	**<0.001**	0.078
**Internalization of thin ideal**								
Baseline	4.13 (1.05)		4.25 (0.76)		3.94 (1.11)			
Post-intervention	3.91 (0.91)^**a,b**^	-0.12 (-0.37; 0.13)	3.84 (0.80)^**a**^	-0.39 (-0.67; -0.12)	3.89 (0.90)^**b**^	0.12 (-0.12; 0.35)	**0.004**	0.045
3 months follow-up	4.09 (1.09)	0.07 (-0.26; 0.40)	4.00 (0.91)	-0.27 (-0.63; 0.09)	3.95 (0.96)	0.04 (-0.27; 0.35)	0.296	0.010

^**a,b**^ The mean difference is significant between groups at the p<0.05 level. The adjusted mean scores are controlled for age and residence type.

^**c**^ p-value and partial-Eta square from random effects analysis of covariance. Partial η2: 0.02 –small, 0.13 –medium, 0.26 –large.

In terms of the sociocultural attitudes towards appearance, the mixed-effects ANCOVA revealed a statistically significant difference between the groups at post-intervention in terms of the impact of the program's resilience against media messages regarding pressure to change the body (*F*_(2, 234)_ = 5.559, *p* = 0.004; partial *η*^2^ = 0.05). The mixed-gender group demonstrated superiority compared with the control group.

#### Body dissatisfaction and drive for thinness

Table 5 describes the change over time in body dissatisfaction and thin ideal internalization in the study groups. The mixed-effects ANCOVA did not reveal any significant changes in the drive for thinness at the post-intervention (*F*_(2, 234)_ = 0.649, *p* = 0.524) or at the follow-up (*F*_(2, 234)_ = 0.100, *p* = 0.904). Body dissatisfaction also did not reveal any significant change at the post-intervention (*F*(_2, 234)_ = 0.957, *p* = 0.385) or at the follow-up (*F*_(2, 234)_ = 0.268, *p* = 0.766).

**Table 5:** Change over time in body dissatisfaction and thin persuasion (raw means [SD] and adjusted means [95% CI]).

Outcome variables	Intervention girls-only group		Intervention mixed group		Control group		Between-group differences ^a^	
		Change from baseline	Raw mean (SD)	Change from baseline	Raw mean (SD)	Change from baseline	*p*	*ES*
	Raw mean (SD)	Adjusted mean (95% CI)		Adjusted mean (95% CI)		Adjusted mean (95% CI)		
**EDI-Body Dissatisfaction**								
Baseline	7.38 (7.14)		6.74 (6.46)		8.43 (8.17)			
Post-intervention	7.43 (6.37)	0.68 (-1.35; 2.71)	7.54 (4.86)	1.85 (-0.37; 4.07)	6.67 (7.12)	0.07 (-1.83; 1.97)	0.385	0.008
3 months follow-up	8.36 (7.47)	0.90 (-1.38; 3.18)	6.98 (5.19)	1.48 (-1.02; 3.98)	8.91 (7.60)	1.70 (-0.43; 3.84)	0.766	0.002
**EDI-Thin Persuasion**								
Baseline	5.35 (5.64)		5.17 (5.02)		5.83 (5.81)			
Post-intervention	5.32 (6.00)	0.27 (-0.95; 1.48)	4.56 (4.76)	-0.63 (-1.96; 0.70)	5.04 (5.81)	-0.25 (-1.39; 0.89)	0.524	0.006
3 months follow-up	4.72 (5.56)	-1.08 (-2.71; 0.55)	4.11 (4.38)	-0.76 (-2.55; 1.02)	5.08 (5.40)	-0.74 (-2.26; 0.79)	0.904	0.001

^**a**^ *p*-value and partial-Eta square from the random effects analysis of covariance. Partial η2: 0.02 –small, 0.13 –medium, 0.26 –large.

#### Body-esteem and body image

The mixed-effects ANCOVA revealed statistically significant changes in the body-esteem appearance subscale and body weight subscale at the 3-month follow-up, with clear dominance of the mixed-gender group (Table 6).

**Table 6:** Change over time in body-esteem and body image (raw means [SD] and adjusted means [95% CI]).

	Intervention girls-only group		Intervention mixed group		Control group		Between-group differences ^c^	
Outcome variables	Raw mean (SD)	Change from baseline	Raw mean (SD)	Change from baseline	Raw mean (SD)	Change from baseline	*p*	*ES*
		Adjusted mean (95% CI)		Adjusted mean (95% CI)		Adjusted mean (95% CI)		
**Body-esteem, appearance**								
Baseline	2.90 (1.04)		2.48 (1.05)		2.74 (0.99)			
Post-intervention	2.85 (1.11)	0.05 (-0.37; 0.26)	2.65 (1.01)	0.38 (0.01; 0.75)	2.83 (1.18)	-0.01 (-0.37; 0.34)	0.231	0.039
3-month follow-up	3.08 (1.20)^**a**^	-0.08 (-0.43; 0.26)	2.90 (1.07)^**b**^	**0.64** (0.24; 1.04)	3.02 (1.00)^**a**^	-0.17 (-0.53; 0.20)	**0.025**	0.088
**Body-esteem, body weight**								
Baseline	2.43 (0.95)		2.28 (0.90)		2.53 (1.10)			
Post-intervention	2.24 (0.99)	-0.16 (-0.45; 0.14)	2.38 (0.71)	0.26 (-0.09; 0.61)	2.52 (1.04)	0.16 (-0.18; 0.50)	**0.089**	0.064
3-month follow-up	2.6 (0.90)^**ab**^	0.05 (-0.28; 0.38)	2.79 (0.81)^**a**^	**0.60** (0.23; 0.98)	2.45 (1.10)^**b**^	-0.24 (-0.58; 0.11)	**0.012**	0.104
**Body-esteem, attribute**								
Baseline	2.20 (0.78)		1.96 (0.88)		2.28 (0.80)			
Post-intervention	1.98 (0.87)	0.11 (-0.28; 0.49)	2.13 (0.88)	0.23 (-0.22; 0.68)	2.18 (0.96)	0.11 (-0.32; 0.55)	0.926	0.002
3-month follow-up	2.16 (0.78)	-0.02 (-0.35; 0.31)	2.35 (0.83)	0.58 (0.21; 0.96)	2.48 (0.83)	0.07 (-0.27; 0.42)	0.093	0.058
**Body image, current**								
Baseline	3.39 (1.35)		3.23 (1.02)		3.29 (1.25)			
Post-intervention	3.32 (1.20)	-0.05 (-0.33; 0.23)	3.15 (0.89)	-0.21 (-0.51; 0.10)	3.38 (1.37)	0.05 (-0.21; 0.32)	0.349	0.010
3-month follow-up	3.50 (1.42)^**a**^	0.23 (-0.12; 0.58)	2.90 (0.98)^**b**^	**-0.53** (-0.91; -0.16)	3.60 (1.33)^**a**^	0.26 (-0.07; 0.58)	**0.003**	0.062
**Body image, ideal**								
Baseline	2.64 (1.21)		2.56 (1.15)		2.29 (0.70)			
Post-intervention	2.53 (0.84)	-0.16 (-0.49; 0.17)	2.30 (0.66)	-0.31 (-0.67; 0.05)	2.42 (0.70)	0.08 (-0.23; 0.39)	0.119	0.021
3-month follow-up	2.66 (0.82)	-0.10 (-0.50; 0.29)	2.78 (1.53)	0.32 (-0.10; 0.74)	2.37 (0.64)	-0.09 (-0.45; 0.28)	0.262	0.014

^**a,b**^ The mean difference is significant between groups at the p<0.05 level. The adjusted mean scores were controlled for age and residence type.

^**c**^ p-value and partial-Eta square from random effects analysis of covariance. Partial η2: 0.02 –small, 0.13 –medium, 0.26 –large.

**Fig 2:**
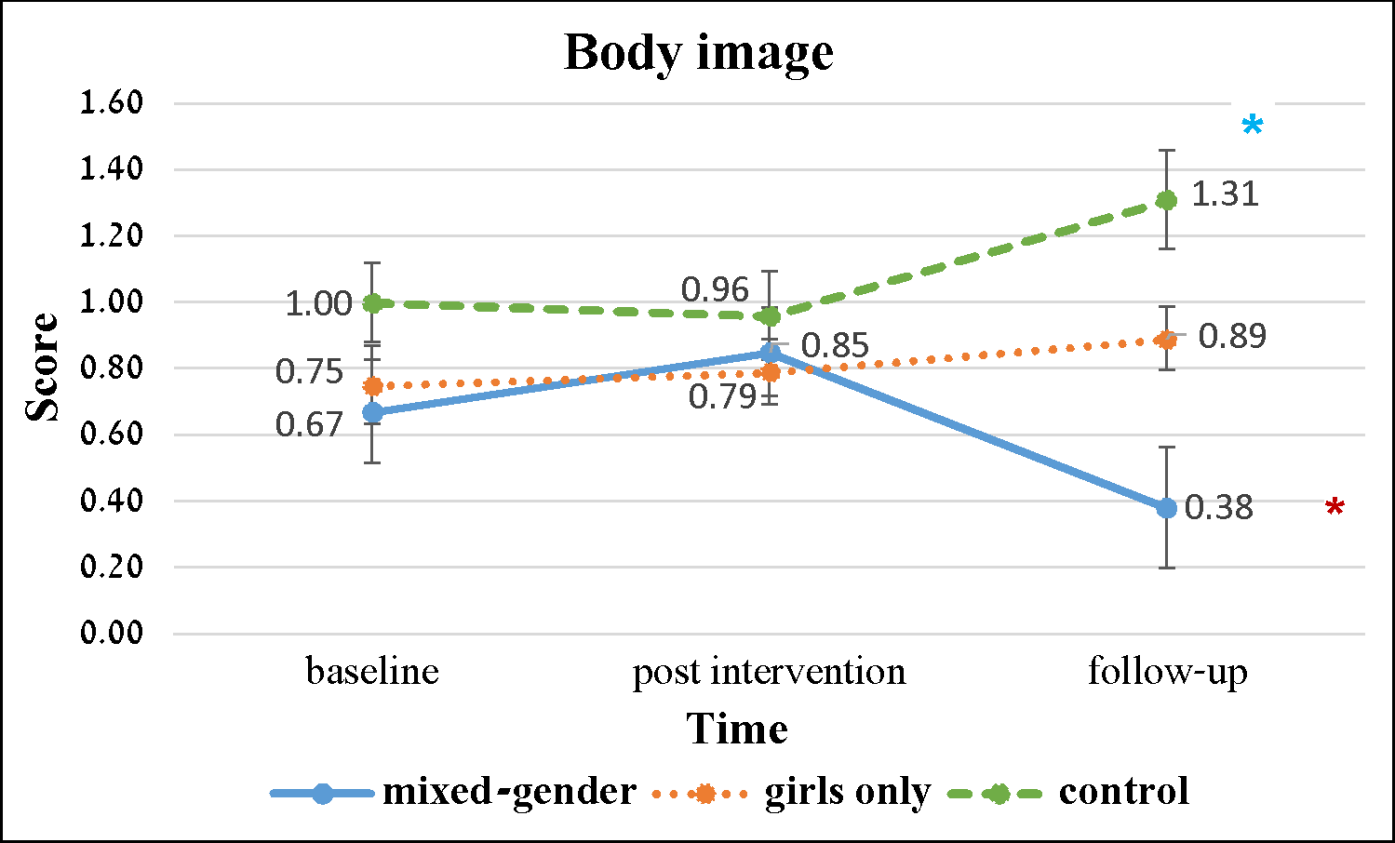
Change over time in the gap between current and ideal body image (mean and SD).

In respect to body image, there was a significant change at the 3-month follow-up in current body image (*F*_(2, 188)_ = 6.178, *p* = 0.003; partial *η*^2^ = 0.062), with a small effect size. In the mixed-gender group, a significant reduction in current body image was observed (positive change), while in the girls-only group and in the control group, there was a significant increase in current body image at the follow-up. Fig 2 shows the changes among the study groups in the gap between current and ideal body image. The gap between current and ideal body image reduced significantly (p<0.05) in the mixed-gender groups, while it increased significantly in the control groups, with no significant change among the participants in the girls’ only groups.

### Mediation models

**Fig 3:**
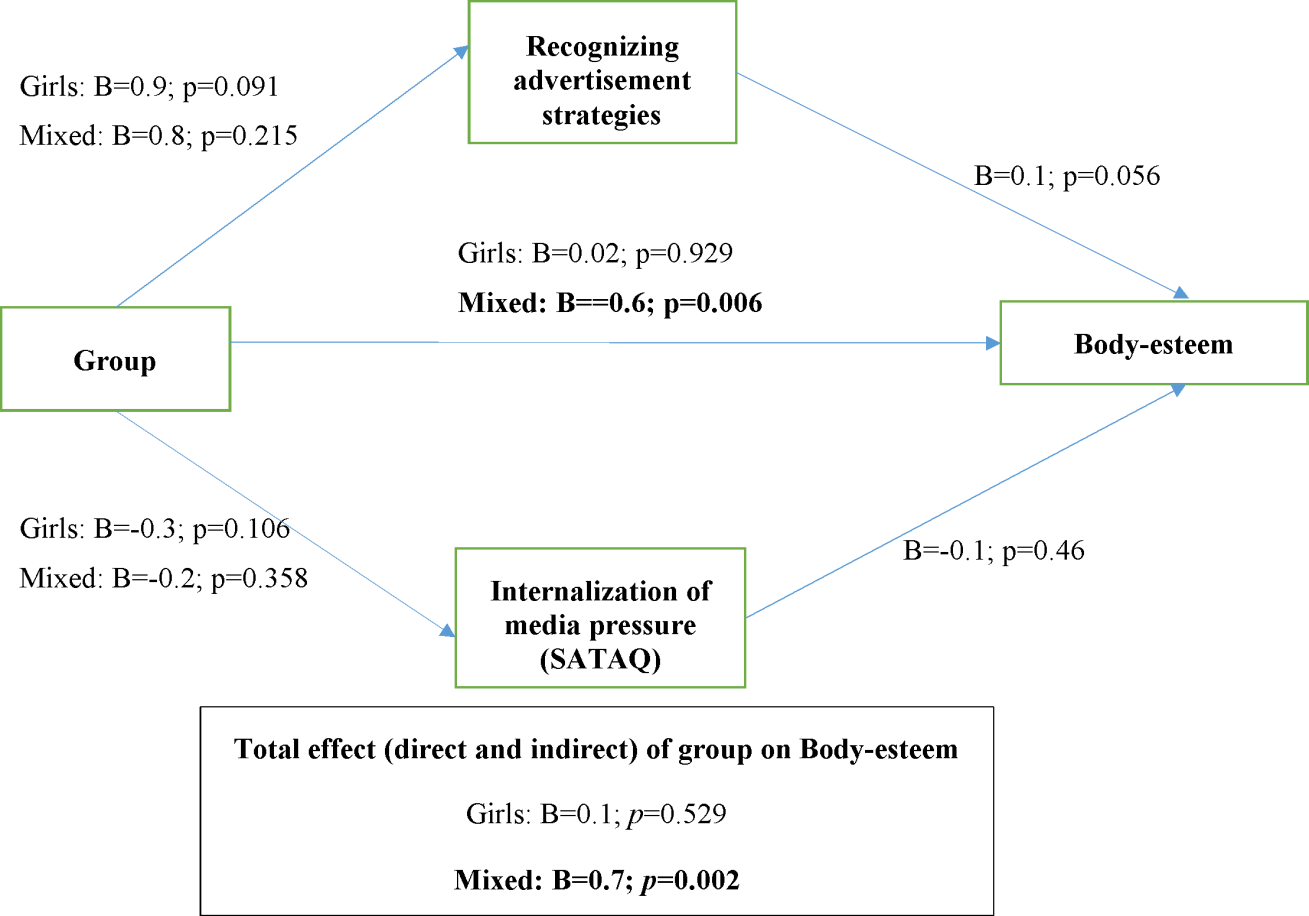
Mediation model for body-esteem (weight subscale).

In terms of body-esteem (the weight subscale), the mediation models revealed that post-intervention, group had a direct effect with p = 0.006 (Fig 3), as well as an indirect effect through changes in media literacy at the follow-up. In the mixed group, the total effect (direct and indirect) of group and media literacy on change in body-esteem at the follow-up was also statistically significant (p = 0.002) compared with the control group, while the effect in the girls-only group was not statistically significant (Fig 3).

**Fig 4:**
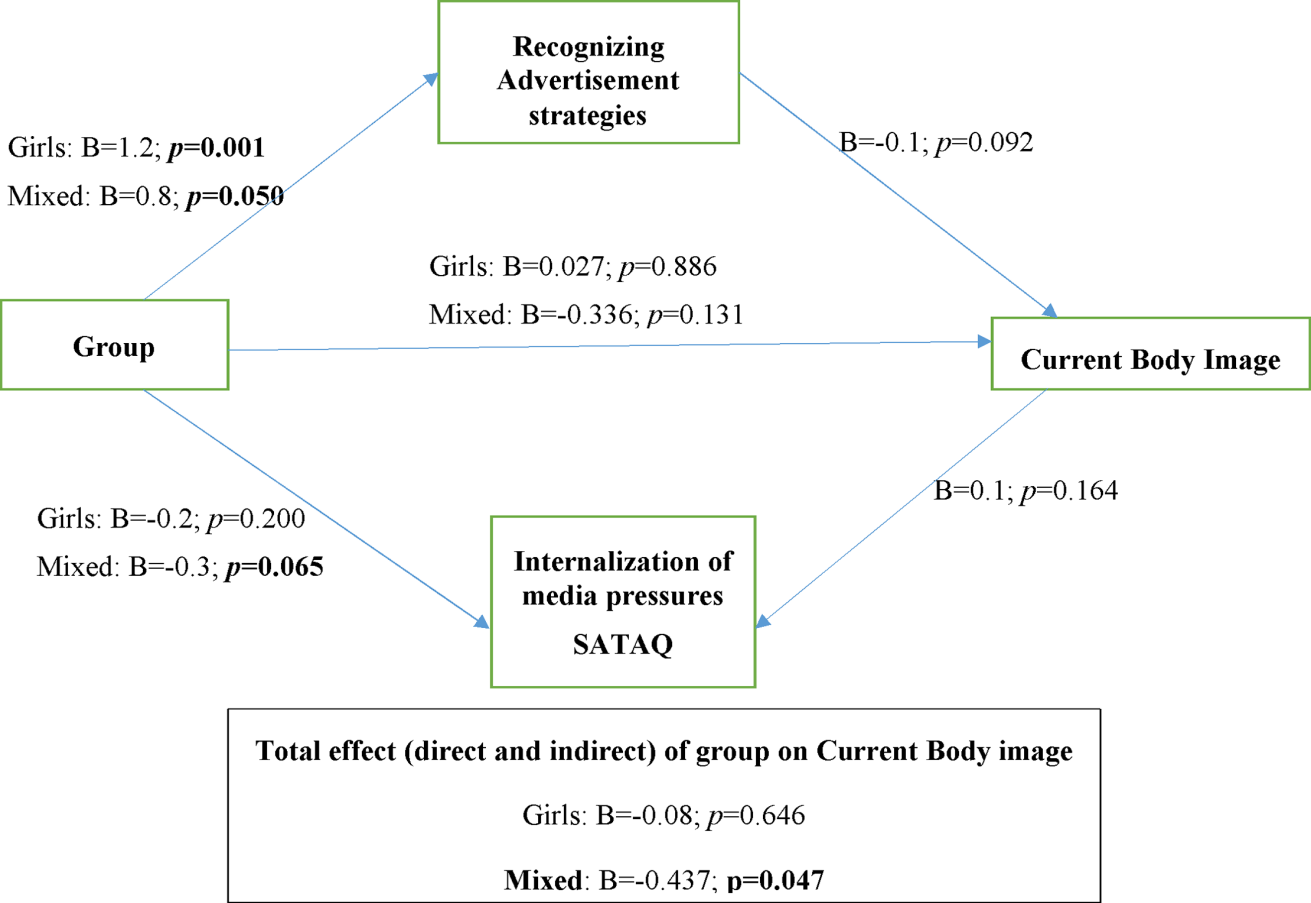
Mediation model for current body image.

Regarding changes in current body image, at the follow-up, only the indirect effect was statistically significant (p = 0.047). This mediation model revealed that post-intervention, within the intervention mixed groups, changes in media literacy (acknowledging advertisement strategies and attitudes due to social pressures) mediated the relationship between group allocation and changes in body image at the follow-up (Fig 4).

#### Programme evaluation

Most of the participants in both the intervention groups (girls-only and mixed-gender) rated their satisfaction with the programme as good or excellent, with satisfaction in the mixed-gender group being slightly higher: 91% evaluated the programme as good or excellent compared with 79% of the participants in the girls-only group.

Regarding attendance rates, a statistically significant difference was found between the intervention groups (*p* = 0.034): 71% of the participants in the mixed-gender group attended all 9 sessions, 27% attended 8 sessions and 2% attended 6 or fewer sessions. In contrast, in the girls-only group, 57% of the participants attended all 9 sessions, 23% attended 8 sessions and 20% attended fewer than 7 sessions (10 times more than that in the mixed-gender group). Overall, higher satisfaction was reported in the mixed-gender group than in the girls-only group.

## Discussion

This cluster-randomized controlled trial was designed to evaluate the impact of ten lessons universal wellness programme administered in girls-only and mixed-gender groups compared with waiting list controls. "In Favour of Myself" is an interactive wellness programme to enhance self-esteem, media literacy and positive body image among adolescents.

To date, there has been no consensus and, therefore, no recommendation for the preferred setting, in terms of gender, for the delivery of universal programmes. Teachers of 13- to 14-year-old adolescents in our country prefer to deliver programmes addressing body image issues to girls-only groups because girls are at higher risk of body dissatisfaction and internalization of thinness. Moreover, girls are considered more emotionally mature and more compliant with discussing these issues [18]. Coping research suggests that compared with boys, girls accept greater responsibility for academic failure, use social support more often and have fewer inappropriate reactions [19].

Regarding the primary research question, at post-intervention, there was evidence indicating the superiority of the mixed-gender groups compared with the girls-only and control groups. The mixed-gender group demonstrated a statistically significant improvement in body-esteem (appearance and body-weight subscales), as well as reduction in perceived current body image and in the gap between current and ideal body image (positive change), compared with the other two groups, which demonstrated lower results or no change, however, the girls-only group improved more than the control group. The mixed-gender group, compared with the control group and the girls-only group, also demonstrated superiority and statistically significant improvement in the internalization of media pressure towards thinness at programme conclusion, achievement that dissolved at the 3-month follow-up assessment. Other prevention programmes, but not all, have also reported significant improvement in the internalization of media pressure [7] [8]. In our study, media literacy improvement was also assessed by studying the change in recognizing the advertisement strategies. Adolescents who participated in the intervention groups (girls-only and mixed groups), as expected, exhibited a statistically significant increase in knowledge at both times of assessment compared with the control group. This finding is in line with a previous meta-analysis and systematic review that observed higher effect sizes for outcomes related to knowledge [3]. Our mediation models revealed a statistically significant direct effect of group (mixed-gender) on body-esteem but only an indirect effect of media literacy on current body image.

The advantage of delivering a wellness programme in a mixed-gender setting can be attributed to the interaction between boys and girls. Because boys usually have a more positive body image than girls [20] and express more self-confidence in their appearance and greater self-esteem, the dialogue between the genders during the sessions might steer the girls towards a more positive view of themselves, and including both genders may contribute to a more complex perspective. It may also help the girls develop greater flexibility and a more conciliatory approach to their bodies, as well as positive self-acceptance, which has been reported by some participants in previous quality assessments of this program. Most published interventions of selected prevention programmes were originally developed for high-risk girls and women [21,6]. Although prevention programmes offered only to females have previously shown to be more effective [22], a programme that includes both sexes has several benefits, as Wiegel et al noted: “First, programs provided only for girls cannot be easily integrated into a typical school programme. Moreover, in spite of the lower prevalence of eating disorders in males, there is strong evidence that eating disorders burden boys in a similar way to girls” [23]. An intervention that addresses both males and females may also promote interaction between boys and girls, thereby helping both genders to see a wider picture when a between-gender misconceptions about attractiveness, thinness and muscularity are addressed. These mediators should be examined in future research.

Competition among participants in the groups, although not measured in our study, must be considered. In a single-gender group, there is greater competition that can be attributed directly or indirectly to lower body image [24,25] and to gender dynamics, which might explain the higher increase in body dissatisfaction and in current body image in the girls-only group in our study. Thus, in our country, to avoid aversive effects of girls-only prevention programmes, the Ministry of Health has advised schools to re-consider their girls-only groups preferences and employ prevention programmes through mixed-gender groups unless they provide reasonable reasons for not doing so. Moreover, girls-only schools are strongly encouraged to add body-image prevention programmes, which are very uncommon in today’s girls-only religious schools.

Nevertheless, most differences in the other variables examined did not reach the level of statistical significance, and the effect sizes were small. The lack of statistically significant differences between mixed-gender and girls-only groups has also been reported by others [7,8]. These small effects are expected, given the floor effects due to starting with a reasonably healthy population.

With respect to self-esteem, we did not find significant change over time in the three study groups, similar to the findings of a previous report about this programme, indicating that we might need more time to demonstrate improvement in this parameter [6]. Other studies that examined the impact of prevention programmes on self-esteem using the Rosenberg questionnaire have reported mixed results. Some studies have found a statistically significant effect at programme conclusion in mixed-gender groups [7, 26], as well as in girls-only groups [8, 27], while others did not observe change in this outcome [28]. This difference in outcomes may be due to the difference in the baseline scores. In our study, the average baseline score was above the reported average of 20.85, however, in studies that indicated improvement, the baseline scores were average and below average [29]. It is well known that programmes have a greater impact for variables that a-priori demonstrate lower/worse status. Other researchers who found significant differences used Harter's [30] self-perception profile (SPPA), which was adapted for adolescents, to examine the change in self-esteem. They reported on positive improvement among girls in mixed-gender groups [31] and in single-gender groups [32]. We did not use this scale due to its length, which is a burden for adolescents when they need to complete additional scales. Future studies should determine the best instrument to assess self-esteem.

In terms of satisfaction with the programme, a higher level of satisfaction with the programme and a higher attendance rate were reported in the mixed-gender intervention group compared with the girls-only group. We attribute this direction to the more appealing and stimulating setting of mixed-gender groups for participants in this age, and less intra-group competition, which is higher among same gender groups, as previously described. To the best of our knowledge, there are no other available publications comparing participant satisfaction among single-gender groups and mixed-gender groups.

Our study provides preliminary evidence, albeit with a small effect size, that delivering universal programmes to mixed-gender groups may contribute to a wellness programme’s effectiveness in enhancing protective factors against social and cultural pressures regarding appearance, a recommendation that has also been suggested by others [33].

### Study strengths and limitations

The present study is a hybrid study of an efficacy and effectiveness trial. Similar to the format of an efficacy trial, which evaluates the efficacy of a programme under optimal conditions (same target population, identical program content, fidelity follow-up and close supervision) and similar to an effectiveness trial, which examines the delivery under natural conditions, in our case, the groups were guided by various facilitators; thus, the results are more easily generalized [34]. However, this design can also be a limitation, as there may be a differential effect of the moderators on the effectiveness of the programme, an effect that should be examined in future studies.

Our sample size was likely too small to detect a large effect size; similar to other reports [7, 35]; unfortunately, the study was limited to the north of the country, where we have only three post-primary schools, thus, our sample size was an *a priori* limitation.

We also tried to address the problem of contamination between the intervention and control groups, however, we could not ensure a lack of contamination because each school provided two intervention classes and one control, and conversation breaks could not be blocked. Nevertheless, the ICC values were in a good range. Longer-term evaluations (12 months or more) could provide information about the maintenance effect of the programme.

## Conclusions

This study provides preliminary evidence of the superiority of mixed-gender groups as the preferred setting for the delivery of a universal school-based wellness programme to promote self-esteem, media literacy and positive body image among adolescents. Further evaluation would ideally include a qualitative study, which should further assess communication that mediates the dynamics between genders to understand the mechanism behind the superiority of a mixed-gender group. Further research with larger sample sizes and longer follow-up assessments is needed to strengthen this assumption and implement it as a policy.

## Supporting information

S1 Fig A flow diagram of the participants. (TIF)

S2 Fig Change over time in the gap between current and ideal body image (mean and SD). (TIF)

S3 Fig Mediation model for body-esteem (weight subscale). (TIF)

S4 Fig Mediation model for current body image. (TIF)

S1 File IRB approval and research protocol. (PDF)

S2 File CONSORT 2010 checklist. (DOC)
