# Supplementary figures and images for: Girls-only vs. mixed-gender groups in the delivery of a universal wellness programme among adolescents: A cluster-randomized controlled trial
Source: PLoS One. 2018 Jun 18;13(6):e0198872. doi: 10.1371/journal.pone.0198872 (PMC6005464; doi:10.1371/journal.pone.0198872)

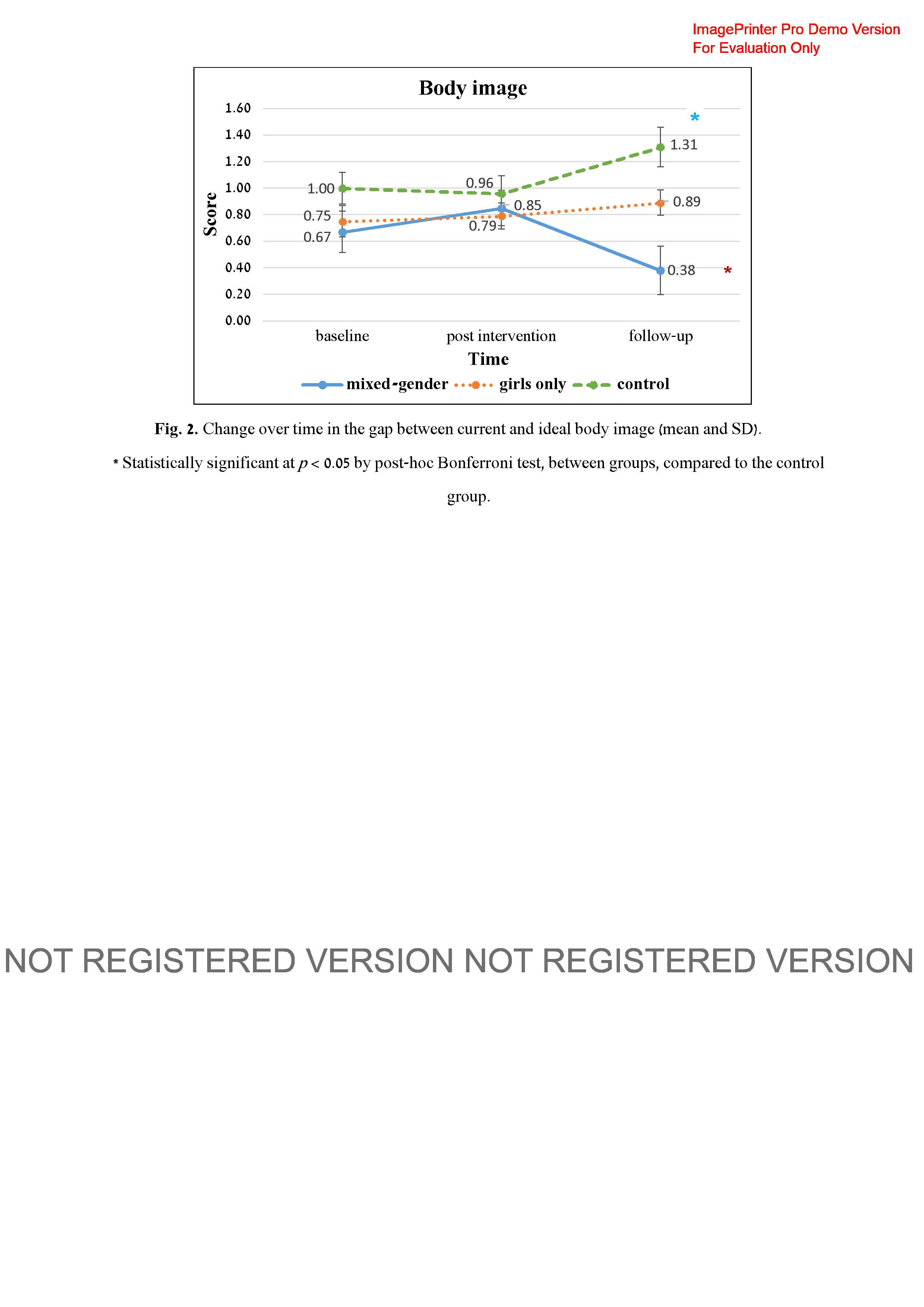

Supplement: S2 Fig — (TIF) [file pone.0198872.s002.tif]

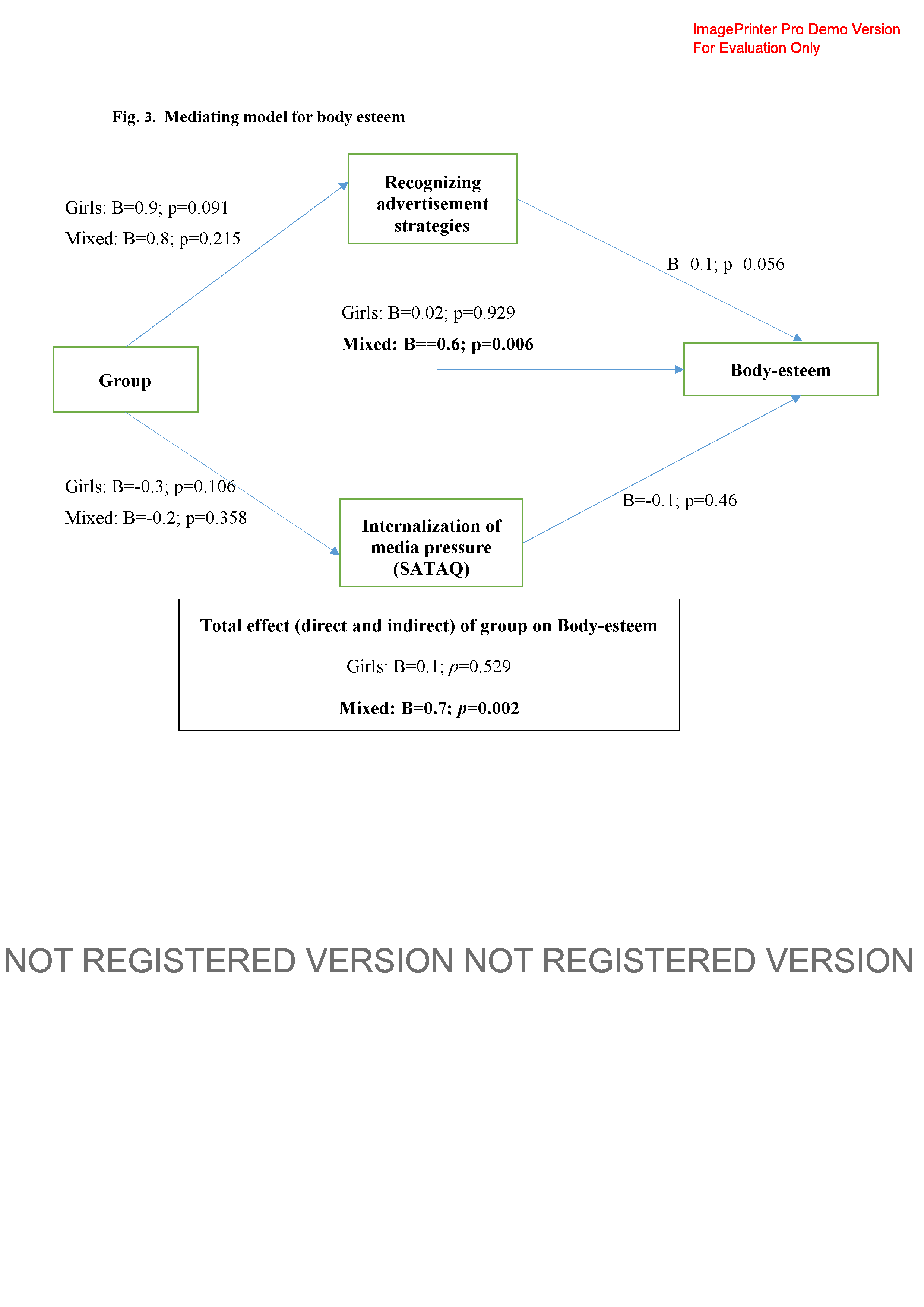

Supplement: S3 Fig — (TIF) [file pone.0198872.s003.tif]

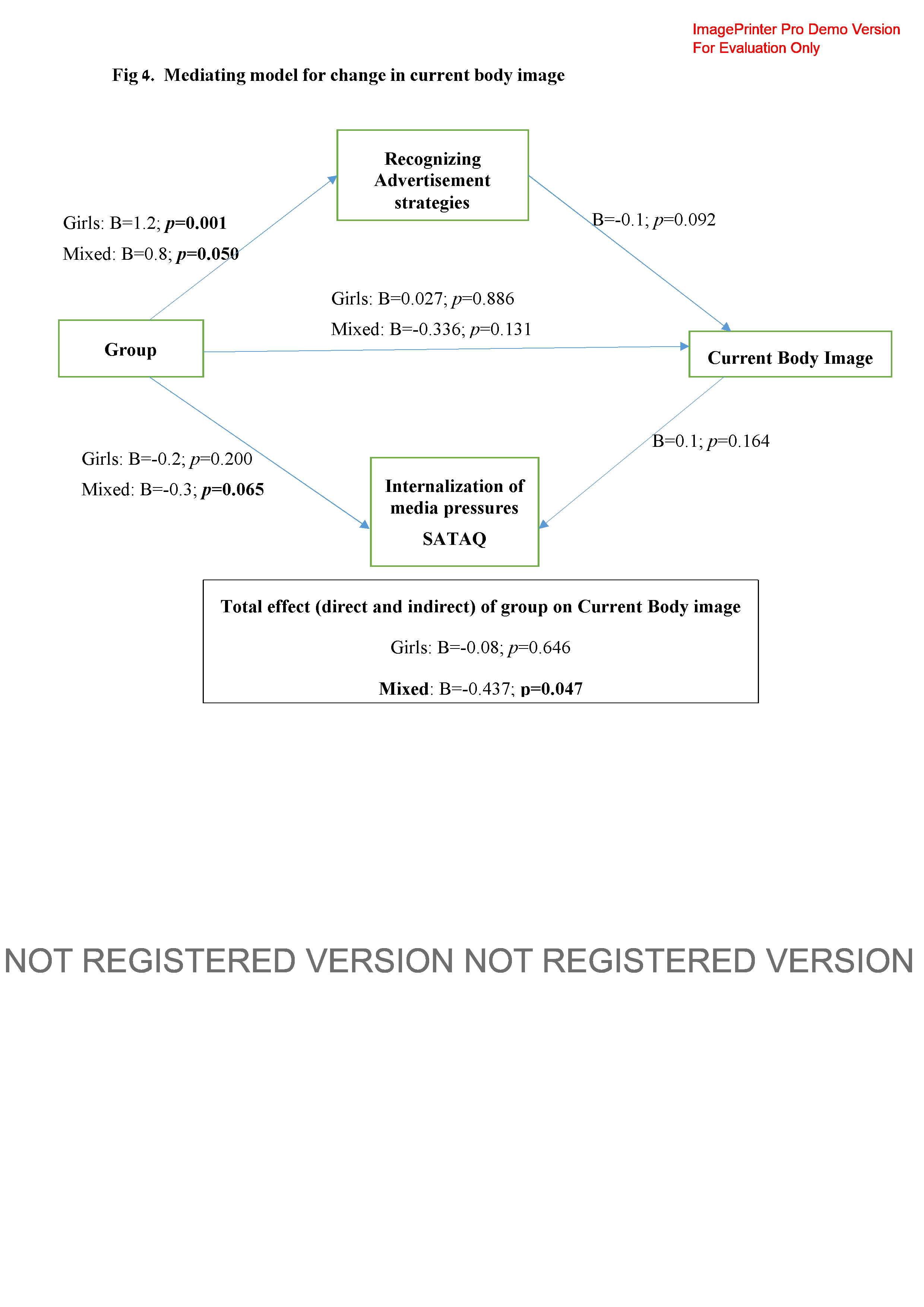

Supplement: S4 Fig — (TIF) [file pone.0198872.s004.tif]
