# Supplementary material for: Girls-only vs. mixed-gender groups in the delivery of a universal wellness programme among adolescents: A cluster-randomized controlled trial
Source: PLoS One. 2018 Jun 18;13(6):e0198872. doi: 10.1371/journal.pone.0198872 (PMC6005464; doi:10.1371/journal.pone.0198872)
Supplement: S1 File — (PDF) [file pone.0198872.s005.pdf]

20.06.2015

לכבוד

ועדת מחקר מוסדית

הנדון: **בקשה לאשר מחקר הבוחן את אפקטיביות התוכנית בעד עצמי חסון שמועברת בהיקף מצומצם בגליל**

בידנו אישור לביצוע מחקר שבוחן את השפעת התוכנית בעד עצמי – תכנית לקדום דמוי עצמי, הערכה עצמית ודמוי גוף חיוביים בקרב בני נוער.

אנו מבקשים אישור להמשיך את המחקר הנ"ל והפעם לבחון את יעילות העברתו בקבוצות של בנות בלבד לעומת קבוצות מעורבות מגדר.

א. הרקע לפיתוח בעד עצמי צעיר ולמחקר הנלווה אליה:

הזהות האישית של הילד, וכן גיבוש עמדות ביחס לאידיאלים חברתיים-תרבותיים, לצד צעדים ראשונים להשתלבות בעולם הבוגר. חלק ניכר מהאתגר בגיבוש הזהות הזו נובע מהשינויים הפיסיולוגיים הקוגניטיביים והרגשיים המתרחשים במהלך תקופה זו ואף לאחריה. רצף השינויים בגוף ובנפש לעתים מוליד קשיים התנהגותיים, ערעור של הזהות העצמית והמינית, מצבי רוח משתנים, חוסר ביטחון עצמי ודמוי גוף שלילי (Harter, 1999).

עם תחילת גיל ההתבגרות, המראה הגופני, המעורבות החברתית והביטחון העצמי הופכים לקריטיים כחלק מבניית הדימוי העצמי של הילד. בחברה בה אנו חיים, הפועלת בחיפזון ומחייבת התבגרות מהירה מאוד מהילד, חוסר סיפוק ודימוי עצמי נמוך מתחילים לצוץ בגילאים מוקדמים יותר ויותר. הקשר בין דימוי עצמי בעייתי לבין תפקוד וסטטוס פסיכולוגי הוכח במחקרים רבים (Clay et al., 2005).

חיפוש הריגושים האופייני לגיל זה, לצד התסכול הנובע מהפער בין האידיאל החברתי ביחס למראה ולסטטוס האישי והחברתי מניעים חלק מהמתבגרים להתנהגויות מסוכנות. בנות עשויות להישאב לדינמיקות של הפרעות אכילה ופעילות גופנית אובססיבית ואילו בנים מועדים יותר לפתח בעיות התנהגות שקשורות להאדרת הדימוי הגברי ומתבטאות בתוקפנות עד אלימות לצד טיפוח שרירניות ו/או שימוש בסטרואידים אנבוליים. לפיכך, מניעת התנהגויות מסוכנות ומזיקות בגיל ההתבגרות הפכה להיות בראש סדר העדיפויות בתחום בריאות הציבור (Gupta, 2011; Pratt & Woolfenden, 2009).

ניתן לחלק את תקופת ההתבגרות ל:.

- **גיל ההתבגרות המוקדם**- גילאי 10-13 . שנים שמאופיינות בצמיחה פיזיולוגית (התגברות אכילה ושינה), שנויים הורמונליים ורגשיים. השינויים הגופניים המהירים גורמים להתעסקות-יתר של המתבגר בדימוי הגוף שלו ובהופעתו החיצונית. לעתים קרובות ההסתגלות לשינויים הפיסיולוגיים וההתמקדות בדימוי הגוף כרוכים בתחושת מצוקה. התחלת מעבר מחשיבה קונקרטית למופשטת והתמקדות ב" עצמי" בהשוואה לאחר. פיתוח עמדות סטריאוטיפיות כחלק מתהליך פיתוח הזהות לצד הבעת ביקורת עצמית ועל אחרים.

את תחילת ההתבגרות מאפיין המעבר מתלות בהורים לעצמאות וזהות עצמית. המתבגר מגלה פחות עניין בפעילויות ההורים, מתרחק מהם בהדרגה ומקדיש את רוב זמנו לפעילות עם בני גילו. ההבשלה הפסיכולוגית של המתבגר כרוכה בתופעות של מרידה בסמכות ההורים, ועל כן קיימים דיווחים על מתח מוגבר ביחסים עם ההורים. מצבי הרוח המשתנים והבלתי צפויים תורמים גם הם למתחים אלו (Mäkinen et al., 2012).

- גיל ההתבגרות האמצעי- 14-16 שנים בהם מתפתחת חשיבה מופשטת ולהט השמור לבני הנעורים ביחס לטיעונים אידיאליסטיים אלו או אחרים, התפתחות יכולת התבוננות על העצמי ("אגו צופה"), פיתוח המוסריות, התנסויות בתפקידים לצד זיהוי רצונות וגבוש עולם ערכי/מוסרי, דתי. בשנים אלו מדווח על ירידה בקונפליקטים עם ההורים והתעניינות בבני המין השני ובמין לצד סיכון מוגבר להתפתחות התנהגויות מזיקות (הפרעות אכילה, סמים, אלכוהול, מתירנות מינית).
- **גיל ההתבגרות המאוחר**- גילאי 17-20 שמאופיין באינטימיות רבה יותר עם אחרים, פחות השפעה של החברים ועצמאות גדולה יותר. נעשים צעדים משמעותיים בתהליך הנפרדות, שלילת התלות ופיתוח העצמאות, תוך הגדרה עצמית והגדרת גבולות העצמי. פיתוח מיניות מלאה יותר התייחסות למשיכה ונטיה מינית וגיבוש זהות מינית. חיפוש ריגושים והתנסויות חדשות. בבנים, שיא הצמיחה הפיסית. התעניינות גוברת בעולם הרחב, עיסוק במוסר עקרוני, מודעות חברתית ואקטיביזם (Ashford et al., 2010).

התנהגויות מזיקות בגיל ההתבגרות

בגיל ההתבגרות ישנה רגישות סביב סוגיות של ערך עצמי ושל ביטחון עצמי. אחוז המתבגרים הסובלים מדימוי עצמי נמוך הינו גבוה. הסקרנות ובדיקת הגבולות האופייניים לגיל ההתבגרות ולתהליך גיבוש הזהות שמתרחש בו, מעוררים במתבגרים רצון להתנסות בכל מה שרק ניתן. הסקרנות מחד והדחפים מאידך, לצד המושפעות הגדולה מחברת השווים והלחצים החברתיים והתרבותיים מגבירים את הסיכון של שכבת גיל זו להתנסות בהתנהגויות מסוכנות ולא אחראיות. מרבית ההתנהגויות וההתנסויות המסוכנות מתגברות על רקע מצוקה רגשית כלשהי וצורך בהרחקה והרגעה לצד רצון ל"שדרוג עצמי" או "תיקון עצמי" על רקע ההתבוננות הביקורתית בעצמי לצד ריבוי ההשוואות לאחר ובמיוחד לדמויות נערצות ומודלים של הצלחה. הטכנולוגיה המודרנית הביאה שעות אינסופיות של צפייה בתקשורת ויזואלית (MTV) ושימוש באינטרנט שמקשר אותנו לעולם אך גם חושף אותנו להתנהגויות מזיקות (Stice et al., 2011;Wilksch, 2014).

החברה כיום משקפת תמונה מעוותת של אידיאל היופי עבור גברים ונשים מכל הגילאים. כאשר מתבגרים צופים בדימויים אלה הם מרגישים נחותים במראה הפיזי שלהם ביחס לאידיאל היופי כפי שמשתקף בתקשורת.

בתרבות המערבית הנוטה לסגוד לרזון, רבים מהמתבגרים מאמצים דפוסי תגובה דרמטיים וקיצוניים כדי להרגיש יותר טוב עם עצמם. מחקרים מדווחים כי 36%-50% מהמתבגרים בגילאי 11-16 לא מרוצים ממבנה גופם או שלפחות פעם אחת בעבר ניסו דיאטה מסוג כלשהו. תוצאות אלה משקפות נאמנה גילאים צעירים אף יותר כתלות ביכולת הנוער לבצע הפנמה של המסרים המשוקפים להם בתקשורת . לחץ וציפיות לא-מציאותיות מצד התקשורת, המשפחה והחברה גורמים למתבגרים להאמין שעליהם לעשות כל שביכולתם על מנת להיראות לפי האידיאל בתקשורת ולא כיצד שהם נראים בפועל (Tiggemann, 2005).

גורמים מגנים מפני התנהגויות מזיקות שעומדים בלב תכניות מניעה

הספרות מתייחסת לגורמים מגנים אישיים, משפחתיים וחברתיים מפני התנהגויות מזיקות. הערכה עצמית גבוהה, דמוי גוף חיובי, אוריינות למסרים בתקשורת המונים (i.e. media literacy), מערך משפחתי וחברתי מיטיב, תחושה של רווחה רגשית (emotional well-being) בנוסף למיומנויות התמודדות מיטיבות וחוסן אישי הוזכרו בספרות כגורמים מגנים מפני התנהגויות מזיקות (Barr et al, 2003; Stice, 2002) .

התכנית בעד עצמי צעיר מתמקדת בשיפור ההערכה העצמית, הדמוי העצמי, דמוי הגוף ואוריינות למסרי המדיה.

- **הערכה עצמית ודמוי עצמי בבני נוער**

דימוי עצמי, הוא האופן שבו האדם תופס את עצמו ואת הערכים איתם מזוהה ביחס לזולתו, בתחומים שונים בחיים. הדימוי העצמי מושפע בין היתר מהערכה עצמית (Bacchini & Magliulo, 2003) .

הערכה עצמית, היא התחושה הכללית של הפרט ביחס לשווי של העצמי . ככל שחוויות ההצלחה שלנו בחיים רבות לעומת הציפיות שלנו ושל החברה מעצמנו כך ההערכה שלנו גבוהה וההיפך (Petersen et al., 1984) . הערכה עצמית חיובית נחשבת כגורם מגן כנגד לחץ מצד בני השווים והשפעת התקשורת ואילו הערכה עצמית נמוכה נחשבת כגורם סיכון שתורם לפגיעות חברתית ורגישות להשפעות חיצוניות כמו לחץ חברתי ולחץ מהמדיה. קיימים דיווחים כי בגיל ההתבגרות קיימת ירידה בהערכה עצמית בקרב המתבגרים כתוצאה מהשנויים הפיסיים , הקוגניטיביים והרגשיים שלהם נתונים והנטייה המוגברת לביקורת עצמית בפרק זה של החיים. השינויים ההתפתחותיים הקשורים לגיל ההתבגרות נוטים לשבש את הרצון של הבנות להתאים עצמן לאידיאל הרזון המקובל חברתית מה שעשוי גם לפגוע בהערכה העצמית שלהן. ההערכה העצמית

של בנות במקרים רבים מתקשרת למראה ומושפעת יותר מפופולריות, הופעה חיצונית, תקשורת בין אישית ודמיון לדמויות נערצות (Neumark-Sztainer et al., 2006).
בבנים לעומת זאת, הסטטוס החברתי הוא גורם מכריע בתפישת ההערכה העצמית, אך גם המראה. כשבנים מתאמצים לאמץ את האידיאל הגברי המאופיין בגוף שרירי וסממנים של גבריות. עבור בנים בגיל ההתבגרות השתתפות בכל סוג של ספורט ופעילות גופנית הייתה קשורה להערכה עצמית גבוהה יותר וספורט נחשב לגורם מגן מפני הערכה עצמית נמוכה.
לעומת זאת, בנים שעסוקים בהגדלת מסת שריר בגילאים אלו נמצאו כבעלי הערכה עצמית נמוכה יותר (Smolak, Michael. Levine, & Thompson, 2001).

- **דמוי גוף חיובי גורם מגן**

דימוי גוף, מבטא את האופן בו חווה, חש ומתייחס הפרט לגופו (Cash, 2004). כבר בגיל 5-7 שנים קיימים דיווחים על ילדות שמביעות חוסר שביעות רצון ממבנה גופן, רצון להיות קט נות יותר ואף שיח על רצון לעשות דיאטה. להבדיל, בנים מגיל 5 ואילך שואפים למבנה גוף משולש: כתפיים רחבות ומותניים צרות, יותר מאשר מבנה גוף רזה או שמן. בנים המצליחים להגיע למבנה הגוף הזה נחשבים כאטרקטיביים יותר וזוכים להטבה ופופולריות חברתית (Grogan, 2007).
בנים בגיל 9-10 שנים שאפו למבנה גוף גדול יותר מגודלם הנוכחי בעוד ילדים בוגרים יותר (גילאים 13-14) שאפו למבנה רזה יותר יחסית לאכן שחוו את מבנה גופם. בנים בגיל 11 היו פחות שבעי רצון ממשקלם יחסית לילדים בגיל 8 וקיים יחס ישר בין תפישת מבנה הגוף והערכה העצמית, במיוחד בקרב בני ה-11. ממצאים אלו מעידים כי ככל שהמתבגר קרב לגיל ההתבגרות, הוא נעשה מודע יותר לאידיאלים ולערכים החברתיים-תרבותיים וחותר לשם. אין זה מפתיע, אם כך, כי אחוז הילדים בגיל טרום-התבגרות הסובל מדימוי עצמי בעייתי הולך וגודל (Thompson & Cafri, 2007).
תחילת גיל ההתבגרות גוררת שינויים גופניים אשר, לרוב, מרחיקים את גוף הנערה מהקונבנציות החברתיות של יופי. נשים צעירות שואפות להיות מושלמות בעיקר ביחס למראה הפיזי שלהן, ומתארות את אידיאל היופי כ"גבוהה, רזה במיוחד, ודקת מותניים". למצער, אידיאל זה אינו אפשרי עבור מרבית הנשים, מה שעשוי לגרום לחוויה של "לא מספיק טובה", ירידה בהערכה העצמית ופגיעה בדמוי העצמי. במקרים מסוימים תהליך זה עשוי אף להתפתח למצוקה רגשית ותגובה קיצונית שמהווה סיכון להתפתחות הפרעות אכילה. בקרב בנים, אידיאל היופי מוגדר כ "כמבנה רחב כתפיים וצר מותניים, מבנה גוף שרירי". הספרות מדווחת כי חצי מהבנים בגיל ההתבגרות שואפים למסת גוף גדולה יותר (משאלה להידמות לאידיאל היופי) בעוד החצי השני שואף למסת גוף קטנה יותר (משאלה לרזות או להישאר "קטן"). חלק ניכר מהנערים והנערות בגיל ההתבגרות וגם הילדים בגילאים הצעירים יותר סובלים מתסכול ביחס לפער בין המראה הפיזי האמיתי שלהם ותפישת הדימוי העצמי שלהם כתוצאה ממסרים אותם הם מקבלים ממקורות שונים כגון התקשורת, ההורים ועמיתים (Yager et al, 2013).

- **אוריינות מדיה (Media Literacy) כגורם מגן מפני השפעת תקשורת המונים.**

אחד מגורמי הסיכון להפרעות אכילה והתנהגויות מזיקות הוא הפנמת מסרים של התקשורת שמעודדים לסגידה אחר אידיאליים חברתיים כגון רזון, שריריות, הנאה, מותרות עד לנקודה שהם הופכים להיות עקרונות נוקשים שמנחים את המתבגרים ואף מנהלים אותם (Thompson et al., 2004).
אוריינות מדיה מייצגת גישת מניעה מבטיחה שנועדה להקטין את הפנמת התקשורת ובכך להפחית את הסיכון להתפתחות של הפרעות אכילה. גישה זו נשענת על התיאוריה ההתנהגותית-קוגניטיבית ועל תיאורית החיסון, על פיהן מנסים להפחית את גורמי הסיכון להפרעות אכילה באמצעות שימוש בפעילויות למידה אשר בונות מיומנויות להתנגד לתפיסה החברתית. גישה זו מתמקדת בעידוד התלמידים לאימוץ הערכה ביקורתית על תכני המדיה כדי שיוכלו לזהות, לנתח, לאתגר ולהציע חלופות לאידיאלים חברתיים ותרבותיים שמוצגים במדיה להמונים (Wilkisch, 2014; Yager et al, 2013).
תכני התכנית בעד עצמי צעיר מתייחסים לשלושת הגורמים המגנים הללו.

מטרות התכנית בעד עצמי:

המטרה הכללית של התכנית בעד עצמי היא לעודד דמוי עצמי ודמוי גוף חיוביים לצד אוריינות מדיה כדי להגן מפני חשיפה למסרים מזיקים בסביבה.

התמות המרכזיות בתכנית בעד עצמי צעיר:

- קידום Self-caring
- אוריינות מדיה Media Literacy

- העצמה והערכה עצמית
- דמוי גוף וההשתנות שלו במהלך גיל ההתבגרות

בגילאים יותר בוגרים התוכנית כוללת גם:

- פרפקציוניזם
- חוסן רגשי
- התמודדות עם בעיות

התכנית בעד עצמי היא תכנית מותאמת גיל Developmentally oriented:

גילאי 10-12 – בעד עצמי צעיר ,כיתות ה'-ו':

דגש על קבלת השונה, מה זה נורמלי , self-caring השוואות ומודלים קיימים, אוריינות מדיה

גילאי 13-15 – בעד עצמי, כיתות ז'-ח':
מי אני וכמה אני מושפע מהאחר, עמידות ללחץ חברתי הן ביחס לדמוי גוף והן ביחס להסתכנות
גילאי 16-17 – בעד עצמי חסון, כיתות ט-י: איך אני ניראה וכמה זה משרת אותי בהתמודדות עם החיים- דגש על חוסן, חוסן לפרסומת, חוסן למידע, חוסן, מה זו גבריות? מה זו נשיות?

פירוט המטרות ונושאי המפגשים בתכנית בעד עצמי "צעיר"

1. **התבוננות על העצמי (מפגש 1)**
2. **קדום דאגה עצמית (שינה, תזונה, וויסות) (מפגש 2)**
3. **קדום שמירה עצמית (טריטוריה, התייחסות לסביבה הוירטואלית) (מפגש 3)**
4. **אוריינות מדיה (מפגש 4)**
5. **הגברת מודעות רגשית והיכרות עם המונח self- talk** (שיום תחושות, זיהוי נקודת המבט של האחר ואמפתיה לאחר, קדום הסתכלות פרספקטיבית enhancing perspective taking) **(מפגש 5)**
6. **חיזוק כישורים חברתיים ובטחון עצמי (אמפתיה לאחר, קבלת השונה, תרגול תקשורת חיובית) (מפגש 6)**
7. **קדום הערכה עצמית (התייחסות להשוואות) וקבלת המגבלות שלי (מפגש 7)**
8. **קדום דמוי גוף חיובי (מפגש 8)**
9. **התיידדות עם הרעיון של מעבר מילדות להתבגרות (מפגש 9)**
10. **פרידה (מפגש 10)**

עקרונות מתודיים בתוכנית לצעירים:

- 10 מפגשים
- משך מפגש 45 דקות בלבד (בניגוד לבוגרים שהוא כפול)
- כל מפגש פותח בחיבור למפגש הקודם כדי להדגיש את עקרון הרצף
- בכל מפגש סרטון + 2 פעילויות משחקיות
- הפעילויות מבוססות על עקרונות של: חברתיות, תחרותיות, אתגר, חוויה, הנאה, ביטוי, גילוי .

עד כה קיימים שני פרסומים שהציגו ממצאים המעידים על בעד עצמי כתוכנית מניעה אפקטיבית . ,.Golan et al) . 2013;2014)

ב. מטרת המחקר וחשיבותו

1. ביצוע מחקר פיילוט להערכת התקבלות התוכנית בעד עצמי צעיר.
2. חקר ראשוני של השפעת התוכנית באמצעות מחקר כמותי ומחקר איכותני.

ג. מהלך עבודה מתוכנן

<u>סוג המחקר:</u> מחקר מבוקר אקראי קבוצתי (Group-Randomized Trials)
<u>אוכלוסיית המחקר:</u> 400 בני נוער בגילאי 13-14
<u>קריטריונים להכללה בקבוצות המחקר:</u> ילדים שהוריהם חתמו על כתב היעדר התנגדות להשתתפות ילדיהם במחקר, הן בקבוצת ההתערבות והן בהשוואה. ילדים שהם עצמם חתמו על כתב ההסכמה המנדטורי שמופיע בראשית השאלון . ילדים שמילאו את השאלונים לפחות פעמים. קבוצות מיקוד כאמצעי למחקר איכותני תתקיימנה בסיום התכנית לקבוצת ההתערבות.

ד. **<u>השערות המחקר:</u>**

א. בקרב משתתפי התוכנית ימצא שיפור ב: הערכה עצמית, אוריינות מדיה ודמוי גוף

ב. שיעור השיפור לאחר 3 חודשים יהיה קטן משיעור השיפור בסיום התוכנית.

ה. **<u>אוכלוסיית המחקר:</u>**

בני נוער בכיתות ו'-ז' מבתי ספר ממלכתיים

ישלח להורים מכתב המסביר את התוכנית, מעדכן ביחס להשתתפות התלמידים במחקר הנלווה, מספק טלפון לפניות ושאלות ומזמין הורים מתנגדים להודיע על כך שמתנגדים להשתתפות ילדם במחקר.

קריטריונים לאי הכללה: לא יכללו במחקר ילדים אשר לא מילאו את השאלון לפחות בשני זמנים שונים (לפני המחקר, בסיומו ו/או 3 חודשים מסיומו).

- **בקשה לאישור הלסינקי מצורפת (נספח א') וכתב הסכמת הורים (נספח ב')**
- **התוכנית הועברה במקביל לשפ"י , אך מפאת חלופי מנהלות שם הפגישה עמם תתקיים רק בחודש יולי. תוכנית המקור של בעד עצמי אושרה על ידי שפ"י.**

ו. **<u>אופן העברת התוכנית</u>**

התוכנית תועבר בבתי הספר במהלך שעות הפעילות הרגילות של בית הספר לקבוצות של 15-20 תלמידים.

התוכנית מועברת על ידי מנחים שהוכשרו לכך מבין תלמידי המכללה האקדמית תל חי ועל ידי יועצות חינוכיות בבתי ספר נבחרים שקיבלו הדרכה מתאימה להעברת התוכנית.

ז. **<u>הערכת התוכנית:</u>**

התלמידים וסוכני השינוי יתבקשו למלא שאלון שפותח עבור תכנית זו ועבר תהליך תיקוף ובדיקת מיומנות, בשלוש נקודות זמן. בתחילת התוכנית, בסיום התוכנית על מנת להעריך את השפעות התוכנית בטווח הקצר וכחצי שנה לאחר סיום התוכנית על מנת להעריך תוצאות בטווח הרחוק, כמו גם שאלון עם פרטים דמוגרפיים.

שלושת השאלונים של כל משתתף יחוברו על ידי רכז הפרויקט באתר הנבחר, ויועברו לידי החוקרים **<u>ללא שמות הילדים</u>**.

השאלון בודק את הפרמטרים הבאים (נספח ג'):

- ידע ביחס לגיל ההתבגרות, דימוי עצמי, דימוי גוף, השפעת סטריאוטיפים והשפעת פרסומות.
- עמדות ותפיסות ביחס לשונה, למראה, להשתנות, להשוואות.
- מדידת שינוי התנהגותי ביחס לתקשורת בין אישית, לצורת האכילה, רמת הפתיחות וליכולת לדבר על נושאים רגישים.
- שינוי בסטאטוס החברתי והאינטראקציה בין חברים.
- שינוי במצב הרוח ובסטטוס החברתי.
- מדידת הערכה עצמית ע"י שאלון רוזנברג.
- מדידת דימוי גוף ע"י הערכת דמויות.
- השאלון נמצא בימים אלו בבדיקה סטטיסטית של התכונות הפסיכומטריות.

בשאלון שפותח נכללו תתי סולמות מתוך שאלונים מתוקפים:

- Rosenberg's Scale for self-esteem (RSE) - מדידת הערכה עצמית ע"י שאלון רוזנברג. שאלון זה, מודד הערכה עצמית כוללת ונמצא בשימוש נרחב. הוא כולל 10 פריטים ויש 4 תשובות אפשריות, בעל סולם ליקרט 1-4,טווח הציונים הוא 10- 40. ככל שהציון יותר גבוה זה מעיד על הערכה עצמית גבוה יותר. מבחן זה הוא בעל אמינות ותקיפות גבוהה. (Rosenberg, 1965)
- שאלון דימוי גוף Contour Drawing Rating Scale (CDRS) מדידת דימוי גוף ע"י הערכת דמויות. מציגים בפני הנבדקים 9 דמויות בטווח גדלים החל מתת משקל לעודף משקל. המשתתפים נשאלים היכן הם חושבים שהם ממוקמים בסקאלה והיכן הם שואפים להיות ממוקמים, מהו לדעתם דימוי הגוף פופולרי ומהו הבריא. ההפרש בין שתי התשובות מעיד על רמת חוסר שביעות רצון מהגוף של המשתתפים, כאשר 100% מעיד על שביעות רצון, ערך שלילי מעיד על הרצון להיות שמן יותר וערך חיובי מעיד על הרצון להיות רזה יותר (Thompson & Altabe, 1991).

- Sociocultural Attitudes Towards Appearance Questionnaire-3 (SATAQ-3) - שאלון זה בודק את ההשפעה הסוציו- תרבותית על דימוי גוף ואכילה מופרעת. מורכב מארבעה תת סולמות: המדיה כמקור מידע, לחץ מהמדיה לשינוי המראה, מידת ההפנמה של המראה האידיאלי המוצג במדיה ומידת הפנמה של הגוף האתלטי כאידיאלי. יש 5 תשובות אפשריות. ככל שהציון גבוה יותר הפנמת אידיאל היופי חברתי שמוצג ע"י המדיה היא גבוה יותר. במחקר זה השתמשנו בתת סולם המודד את מידת ההפנמה של המראה האידיאלי המוצג במדיה (Markland & Oliver, 2008).
- Eating Disorders Inventory – 2 (EDI-2) - מודד סימפטומים וגורמי סיכון להפרעות אכילה. כולל 11 תת סולמות: השאיפה לרזון, בולימיה, חוסר שביעות רצון מהגוף, חוסר יעילות, פרפקציוניזם, חובר יכולת לסמוך על אחרים, מודעות עצמית, בגרות הפחדים, וויסות, ביטחון חברתי, סגפנות. אנו נשתמש במחקר זה בשני תת סולמות: שאיפה לרזון וחוסר שביעות רצון מהגוף (Garner, 1991).
- Body Esteem Scale- Mendelson שאלון דיווח עצמי, נוח לשימוש ונחשב כלי מהימן לבדיקת דימוי גוף במתבגרים ב- 3 מובנים: תחושה כללית כלפי המראה, שביעות רצון ממשקל הגוף וייחוס ההערכה העצמית לאחר. השאלון מכיל 23 פריטים. לשאלון תכונות פסיכומטריות טובות, ערך אלפא קרונבך הוא 0.81 -0.95 (Mendelson, Mendelson, & White, 2001)

ח. ניתוח סטטיסטי:

הניתוח הסטטיסטי יעשה בעזרת תוכנת SPSS ל- Windows. ההבדלים במשתנים התלויים לפני ההתערבות ובין רמתם לאחר ההתערבות יבדקו באמצעות מבחני t למדגמים תלויים. ניתוחי ANCOVA Univariate Analysis of Covariance) ו-MANCOVA (Multivariate Analysis of Covariance) ישמשו להשוואת הקבוצות בשינויים שחלו לפני ואחרי ההתערבות, כאשר בכולם המשתנה התלוי יהיה השינוי במדדים השונים שיבדקו לאחר תכנית ההתערבות. משתנה ה- Covariate יהיה המדידה לפני ואחרי ההתערבות והמשתנה הבלתי תלוי יהיה אם בוצעה התערבות או לא.
ניתוחי ANCOVA ו- MANCOVA רב כיווניים ישמשו לבדיקת האינטראקציה בין מספר המשתנים הבלתי תלויים, ההבדל בין הקבוצות במשתנים האיכותיים והדיכוטומיים יבדקו באמצעות מבחני חי בריבוע. ערכים של $p<0.05$ יחשבו כמובהקים מבחינה סטטיסטית. בכדי לבדוק האם קיים קשר בין משתנים (קשר בין גיל המשתתפים למידת השפעת התוכנית) יחושבו מתאמי פירסון. גורמי מנבאים להצלחת התוכנית יבחנו באמצעות רגרסיה הירככית.

נודה על אישור עקרוני **לקיום המחקר** הנלווה לתוכנית בקרב תלמידי חטיבות הביניים בחיפה ובגליל.

בברכה

פרופ' מוריה גולן

ביבליוגרפיה

Ashford, J.B., LeCroy C.W., Lortie K.L. *Human behavior in the social environment: A multidimensional perspective.* Cengage Learning; 2009:720.

Bacchini, D., & Magliulo, F. (2003). Self-image and perceived self-efficacy during adolescence. *Journal of Youth and Adolescence, 32*(5), 337-349.

Barr, C.T., Bryson, S.W., Altman, T.M., & Abascal, L. (2003). Risk factors for the onset of eating disorders in adolescent girls: Results of the McKnight longitudinal risk factor study. *American Journal of Psychiatry, 160*(2), 54-248.

Cash, T.F. (2004). Body image: past, present, and future. *Body Image*, 1(1), 1–5.

Clay, D., Vignoles, V.L., & Dittmar, H. (2005). Body image and self esteem among adolescent girls: Testing the influence of sociocultural factors. *Body Image*, *15*(4), 451–477.

Crocker, J., Luhtanen, R. K., Cooper, M. L., & Bouvrette, A. (2003). Contingencies of self-worth in college students: theory and measurement. *Pers Soc Psychol, 85*(5), 894.

Harter, S. (1993). Causes and consequences of low self-esteem in children and adolescents. In R. Baumeister (Ed.), *Self-Esteem SE - 5* (pp. 87–116). Springer US. doi:10.1007/978-1-4684-8956-9_5

Golan M., Hagay N., Tamir S. (2013). The effect of “In Favor of Myself”: preventive program to enhance positive self and body image among Adolescents. *PLoS One*, 8(11), e78223.

Golan, M., Hagay, N., & Tamir, S. (2014). Gender related differences in response to “In Favor of Myself” wellness program to enhance positive self & body image among adolescents. *PloS One*, *9*(3), e91778.

Grogan, S. (2007). Body image: Understanding body dissatisfaction in men, women and children. New York: Routledge.

Gupta, C. (2011). *The Relation between Body Image Satisfaction and Self-esteem to Academic Behaviour in Adolescents and Pre-adolescents*. University of Manitoba.

Mäkinen, M., Puukko-Viertomies, L.-R., Lindberg, N., Siimes, M. a, & Aalberg, V. (2012). Body dissatisfaction and body mass in girls and boys transitioning from early to mid-adolescence: additional role of self-esteem and eating habits. *BMC psychiatry*, 12, 35.

Markland, D., & Oliver, E. J. (2008). The sociocultural attitudes towards appearance questionnaire-3: a confirmatory factor analysis. *Body image, 5*(1), 116–121

Neumark-Sztainer, D., Levine, M. P., Paxton, S. J., Smolak, L., Piran, N., & Wertheim, E. H. (2006). Prevention of body dissatisfaction and disordered eating: What next? *Eating Disorders*, *14*(4), 265–285.

Petersen A.C., Schulenberg J.E., Abramowitz R.H., Offer D., & Jarcho H.D. (1984). A self-image questionnaire for young adolescents (SIQYA): Reliability and validity studies. *Journal of Youth and Adolescence*, *13*(2), 93–111.

Pratt, B.M., & Woolfenden, S. (2009). Interventions for preventing eating disorders in children and adolescents (Review), Retrieved from http://www.essstörungen argau.ch/media/archive1/ fachpersonen/praevention/ epidemiologie/PrevEDCochrane2002.pdf.

Rosenberg, M. (1965). Society and the adolescent self-image. *Princeton, NJ: Princeton university press.*

Tiggemann M. (2005). Body dissatisfaction and adolescent self-esteem: prospective findings. *Body Image*, 2(2), 129–35.

Thompson, J. K., & Altabe, M. N. (1991). Psychometric qualities of the figure rating scale. *Int J Eat Disord*, *10*(5), 615–619.

Thompson, J.K., & Cafri, G. (2007). The muscular ideal: Psychological, social, and medical perspectives. *Washington, DC, US: American Psychological Association*.

Thompson, J.K., van den Berg, P., Roehrig, M., Guarda, A.S., & Heinberg, L.J. (2004). The sociocultural attitudes towards appearance scale-3 (SATAQ-3): development and validation. *The International Journal of Eating Disorders*, *35*(3), 293–304. doi:10.1002/eat.10257

Yager Z., Diedrichs P.C., Ricciardelli L.A., Halliwell E. What works in secondary schools? A systematic review of classroom-based body image programs. *Body Image*. 2013;10(3):271–81.

Wilkisch S.M. (2014).Where did universal eating disorder prevention go? E*ating Disorders: The Journal of*

Treatment & Prevention, 22:2, 184-192

<u>נספח א': בקשה לועדת הלסינקי</u>

בקשה לאישור ועדת אתיקה לביצוע מחקר בבני אדם[1]

תאריך: 23.6.2015

שם החוקר/ת:פרופ' מוריה גולן

<u>שם המחקר</u>: בעד עצמי –תכנית לקדום דמוי עצמי ודמוי גוף חיוביים בקרב מתבגרים בתחילת ובאמצע גיל ההתבגרות השפעת ה- setting

<u>המשתתפים:</u>

1. **מספר הנבדקים/המשתתפים**:	400

2. **טווח גיל:** 13-14 קטינים (מתחת לגיל 18) – **נדרש טופס הסכמת הורים**/בוגרים

3. האוכלוסייה: [X] ילדי בית ספר עממי וחטיבת ביניים

4. **הדרך להשגת השתתפותם** (הסבר מפורט):

פניה לבתי הספר להשתתפות במחקר קליני מבוקר עם חלוקה אקראית לקבוצת מחקר וביקורת – בדומה למה שנעשה על ידי קבוצת המחקר שלנו בשנים קודמות.

II. שיטות - סמן את <u>כל</u> האפשרויות – <u>כן</u> או <u>לא</u>

		<u>כן</u>	<u>לא</u>
1	העברת שאלונים	X	
2	סקר אנונימי		
3	ראיון		
4	תצפית		
5	הקלטה בוידיאו, צילום, כד'		
6	הקלטה בקלטת שמע		
7	שימוש במסמכים או נתונים קיימים		
8	התערבות קלינית שבוחנת תכנית מניעה	X	

[1] מתוך הועדה להערכת מחקרים בבני אדם, אוניברסיטת חיפה.

III. האם המחקר כולל?

		כן	לא
1	הולכת שולל או הסבר הלוקה בחסר		X
2	איסוף מידע רגיש		X
3	חשיפה לגירויים היכולים להתקבל כמאיימים, מעליבים, מעוררי חרדה, מעוררי זכרונות טראומטיים או כדומה		X
4	חשיפה לגירויים פיזיים כגון: דרגות גבוהות של רעש או גירויים ויזואליים החורגים ממידת הגירוי היומיומי או כאב		X
5	איסוף מדדים ביולוגיים ו/או פיזיולוגיים (דם, רוק, דופק, לחץ דם, מדדים פיזיולוגיים אחרים).		X
6	שימוש בתרופות (נא לתאר את התרופות ואת האמצעים הננקטים לשמירה על בטחון המשתתפים)		X
7	מאמץ פיזי מעבר למקובל ביום-יום (נא לתאר את המשימה ואת האמצעים הננקטים להגנה על המשתתפים)		X
8	סיכון חברתי, משפטי, בראותי, כלכלי או אחר לנחקרים (כגון יצירת סטיגמה, סיכון סטטוס תעסוקתי או הפללה של נחקרים)		X
9	**פניה למשתתפים דרך גורם סמכותי** (מורים מטפלים מעבידים)	X	
10	**תגמול כספי, מתן ציון או אמצעים אחרים לעידוד ההשתתפות**<br>**(נא לתאר)** הילדים יקבלו מתנה סמלית (בקבוק, כפכפים, צמיד סיליקון) בתמורה לזמן שהשקיעו במילוי כל אחד מהשאלונים	X	

II. האם המכתב (טופס הסכמה מדעת) למשתתפים הפוטנציאליים כולל?

		כן	לא
1	תאור המחקר, שמו ומטרתו	X	
2	תועלת המחקר	X	
3	סיכויים ו/או סיכונים לנחקר	X	
4	המשימות המוטלות על הנחקר	X	
5	הצהרה על השתתפות רצונית וזכות המשתתפים לפרוש מהמחקר בכל עת ללא השלכות שליליות	X	
6	הבטחת סודיות, אנונימיות ופרטיות	X	
7	שם החוקר/ת וטלפון או דרך אחרת להתקשר עמו/ה	X	
8	טופס הסכמה להשתתפות רצונית	X	

III. הגנה על המשתתפים

	כן	לא
1. האם טופסי ההסכמה להשתתפות רצונית ישמרו בנפרד מתוצאות הנבדקים?	X	
2. האם תפורסם זהות המשתתפים?		X

3. **מלבד החוקר, האם יוכלו גורמים נוספים לעיין בתוצאותיהם של המשתתפים**? סטטיסטיקאי ותלמידות המוסמך המחקרי

X

4. האם המחקר כולל סיכונים ו/או השלכות שליליות אפשריות על הנחקרים? אם כן, אילו אמצעים יינקטו על מנת להפחית את הסיכון לתוצאות כאלה **לא כולל סיכונים**

5. במקרה של נזק לנחקר, אלו דרכי התערבות ינקטו? **לא רלבנטי**

6. נא לתאר את האמצעים אשר ינקטו להגן על זהות המשתתפים, והנתונים המתקבלים

לאחר שכל ילד מילא 3 שאלונים הם יחוברו למקבץ אחד ויועברו על ידי עוזרת המחקר של התוכנית אל הסטטיסטיקאי. הסטטיסטיקאי יקבל קובץ משותף ללא שמות הילדים ויעביר את הממצאים של העבוד הסטטיסטי לתלמידת המחקר אליה הוא משתייך.

הנני מצהיר/ה בזאת שהמידע הנ"ל מלא נכון ומדויק והצעת המחקר תואמת את הסטנדרטים הבינלאומיים והאוניברסיטאיים לניהול מחקר אתי.

שם החוקר/ת: פרופ' מוריה גולן

המכללה האקדמית תל-חי
למדע, סביבה וחברה

<u>נספח ב'</u>: כתב היעדר התנגדות הורים

הורים יקרים,

ילדכם ישתתף בשנה הקרובה בתוכנית "בעד עצמי" במסגרת בית הספר בו הוא לומד. את התוכנית מלווה מחקר שמעריך את יעילותה **באמצעות שאלון ממוחשב** אותו ימלאו הילדים לפני ואחרי התוכנית.

אנו מבקשים הסכמתכם להשתתפות ילדכם במחקר זה.

מצורף הסבר מפורט על התוכנית והמחקר כך שתוכלו לתת את **הסכמתכם מדעת** להשתתפות ילדכם בו.

1. <u>כללי</u>

מטרת כל מחקר מדעי היא להרחיב ולפתח את הידע הקיים, כדי לנסות למנוע מצבי תחלואה פיסית ו/או נפשית. המחקרים הרפואיים בבני אדם מקבלים אישור על פי החוק, רק אם הם מגנים על זכויות המשתתפים לרבות פרטיותם. המחקר, בו ילדכם מתבקש להשתתף, קיבל אישור שכזה. חשוב לנו שתבינו את פרטי המחקר ומטרותיו, כדי שהסכמתכם להשתתפותו תינתן מתוך ידיעה והבנה.

2. <u>המחקר: הערכת התוכנית "בעד עצמי"</u>

תיאור המחקר: התוכנית בעד עצמי תוערך באמצעות מלוי שאלונים על ידי התלמידים בשלושה מועדים: לפני תחילתה, בסיומה ולאחר חצי שנה. השאלונים כוללים שאלות המתייחסות לשביעות הרצון מהתוכנית על חלקיה השונים, שאלות ביחס לידע ולעמדות הקשורות לדמוי עצמי, השפעת המדיה בכלל ופרסומות בפרט, תקשורת חיובית, דמוי גוף ודמוי עצמי.

המוסד באחריותו נערך המחקר: המכללה האקדמית תל חי **החוקרת הראשית** במחקר: פרופ' מוריה גולן.

3. <u>המידע</u>

ההשתתפות במחקר כרוכה במלוי שאלונים שיועברו לפני ואחרי התוכנית. החוקרים רשאים לעשות שימוש במידע שניתן בשאלונים **לצורכי מחקר זה בלבד**. לאחר שכל ילד מילא 3 שאלונים הם יחוברו לערכה אחת, יועברו לקובץ משותף ללא שמות הילדים. הקובץ המשותף <u>ללא שמות הילדים</u> ישלח לעבוד סטטיסטי.

במידה וישנן שאלות בנושא נשמח לעמוד לרשותכם במייל shaniaboutboul@gmail.com או בטלפון 0545808814

במידה ולא תתקבל תגובה על מכתב זה אנו רואים בכך הבעת הסכמה להשתתפות ילדכם הן בתוכנית והן במילוי שאלון שנילווה אליה לפני ואחרי.

- **לכל שאלה המתעוררת אתם מוזמנים לפנות טלפונית לעוזרת המחקר: אבוטבול שני 054-5808814**

במידה והנכם מתנגדים שילדכם ישתתף במחקר שמעריך את יעילות התוכנית , אנא מלאו את הספח שלהלן והעבירו אותו להנהלת בית הספר לא יאוחר מיום ______________.

עורכת המחקר: פרופ' מוריה גולן

****

****לכבוד: **פרופ' מוריה גולן**, מכללת תל חי

הנדון: *הבעת התנגדות להשתתפות בני/בתי במחקר*

אני מביע/ה את התנגדותי להשתתפות בני/בתי______________________ (שם הבן/בת)ב המעריך את התכנית "בעד עצמי חסון".

ולראיה באתי על החתום

**תאריך**	**שם ההורה (שם מלא)**	**חתימה**

נספח ג' – שאלון המחקר

כתב הסכמה מנדטורי של התלמיד(רק במקרים של סימון הסכמה, יפתח המשך השאלון).

אני החתום/ה מטה תאריך ________________ ___________________

**שם ושם משפחה**	**בית ספר וכיתה**

1. **התבקשתי להשתתף במלוי שאלונים כחלק ממחקר. אני מבין שהמידע שייאסף ישמש לצורך מחקר בלבד ומהרגע שמילאתי את שלושת השאלונים, ישמט שמי וכל הפרטים המזהים האחרים מגליון הנתונים. הצוות החוקר יקבל את גליון הנתונים ללא הפרטים המזהים שלי.**
2. **הובהר לי כי השתתפותי היא רשות ולא חובה, וכי אהיה רשאי שלא להשתתף או להפסיק את השתתפותי בכל שלב של המחקר.**
3. **לאחר שהוסבר לי כל האמור לעיל, הנני מביע/ה את הסכמתי מרצוני הטוב והחופשי להשתתף במחקר (סמן את התשובה המתאימה) :**
   - כן
   - לא

בעד עצמי– שאלון

פרטים אישיים: (הקיפו בעיגול)

שם ושם משפחה:______________________ **כתובת דואר אלקטרוני:**______________________

שם בית הספר:__________________________ **כיתה:**__________ **מגדר:**1. בן 2. בת

תאריך לידה: ____________ **גיל:**____________________

גדלתי במשפחה:1. הורים נשואים 2. הורים גרושים או פרודים 3. משפחה חד הורית 4. אחר __________

יחס לדת: 1. דתי/ה 2. מסורתי/ת 3. חילוני/ה

מספר אחים (כולל אותך)____________

מקומך בסדר הלידה:1.בכור/ה 2. אמצעי/ת 3. הצעיר/ה ביותר 4. יחיד/ה

צורת מגורים:1. עיר 2. מושב/ה 3. קיבוץ 4. יישוב קהילתי

השכלת האב: (הקיפו בעיגול)

1. בית ספר יסודי
2. בית ספר תיכון
3. השכלה מקצועית
4. תואר ראשון
5. תואר שני ומעלה

השכלת אם: (הקיפו בעיגול):

1. בית ספר יסודי
2. בית ספר תיכון
3. השכלה מקצועית
4. תואר ראשון
5. תואר שני ומעלה

מצב כלכלי (סמנו את התשובה המתאימה): 1. חיים ברווחה גדולה 2. חיים בנוחות 3. חיים בצמצום 4. חיים בדוחק

1. סמנ/י את כל המילים שלדעתך מתארות את הטקטיקות/תחבולות בהן משתמשים המפרסמים?

1. הגזמה 2. אשליות 3. פנייה אל ההיגיון 4. פנייה אל הרגש 5. רומנטיזציה 6. אידיאליזציה 7. אמיתיות, ריאליות 8. הפחדה

**2. כשאת/ה רואה תמונות של אנשים בטלוויזיה, בעיתונים ובפרסומות באיזו מידה זה גורם לך לרצות (סמנ/י את התשובה המתאימה עבורך):**	כלל לא	לעיתים רחוקות	לעיתים קרובות	תמיד
א. לתקן את עצמך	4	3	2	1
ב. לשנות את משקלך	4	3	2	1
ג. לעשות משהו ביחס למראה החיצוני	4	3	2	1
**3. ביחס לעצמי (סמנ/י את התשובה המתאימה עבורך):**	כלל לא	לעיתים רחוקות	לעיתים קרובות	תמיד
א. אני מרגיש/ה אדם בעל ערך, לא פחות מאחרים	3	2	1	0
ב. אני מרגיש/ה שיש לי כמה תכונות טובות.	3	2	1	0
ג. בסך הכול, אני נוטה להרגיש שאני כישלון.	3	2	1	0
ד. אני מסוגל/ת לעשות דברים לא פחות מאנשים אחרים	3	2	1	0
ה. אני מרגיש/ה שאין לי יותר מדי על מה להתגאות	3	2	1	0
ו. יש לי גישה חיובית כלפי עצמי	3	2	1	0
ז. באופן כללי, אני מרוצה מעצמי	3	2	1	0
ח. הלוואי ויכולתי לכבד את עצמי יותר .	3	2	1	0
ט. אני מרגיש/ה חסר/ת ערך לפעמים	3	2	1	0
י. לפעמים אני חושב/ת שאין בי שום דבר טוב.	3	2	1	0
**4. ביחס למראה - עד כמה את/ה מסכים/מה עם המשפטים הבאים:**	כלל לא	לעיתים רחוקות	לעיתים קרובות	תמיד
א. הייתי שמח/ה אם הייתי יכול/ה לשנות את המראה שלי	4	3	2	1
ב. המראה שלי משפיע על הביטחון העצמי שלי	4	3	2	1
ג. אם המשקל שלי היה שונה הייתי מאושר/ת יותר	4	3	2	1
ד. אם הגובה שלי היה שונה הייתי מאושר/ת יותר	4	3	2	1
ה. אם מידת הבגדים שלי הייתה שונה הייתי מאושר/ת יותר	4	3	2	1

5. **ביחס להשפעת התקשורת - עד כמה את/ה מסכים/ה עם המשפטים הבאים (סמנ/י את התשובה המתאימה עבורך):**	בהחלט לא מסכים	לרב לא מסכים	ניטרלי	לרב מסכים	תמיד מסכים
א. לא אכפת לי אם הגוף שלי דומה לזה של האנשים בטלוויזיה או לא	5	4	3	2	1
ב. אני משווה את הגוף שלי עם זה של אנשים בטלוויזיה	5	4	3	2	1
ג. הייתי רוצה שהגוף שלי יראה כמו של הדוגמניות/דוגמנים שמופיעים בעיתונים	5	4	3	2	1
ד. אני משווה את המראה שלי עם זה של כוכבי סרטים וטלוויזיה	5	4	3	2	1
ה. הייתי רוצה שהגוף שלי יראה כמו זה של האנשים בקולנוע/בסרטים	5	4	3	2	1
ו. אינני משווה את הגוף שלי לגוף של האנשים שמופיעים במגזינים	5	4	3	2	1
ז. הייתי רוצה להיראות כמו המודלים שבקליפים	5	4	3	2	1
ח. אני משווה את המראה שלי למראה של אנשים במגזינים	5	4	3	2	1
ט. אני לא מנסה להיראות כמו האנשים בטלוויזיה	5	4	3	2	1

6. הצלליות הבאות מייצגות דמויות שונות. אנא בחרי מתוכן את הדמויות הרלבנטיות עבור ארבע השאלות הבאות

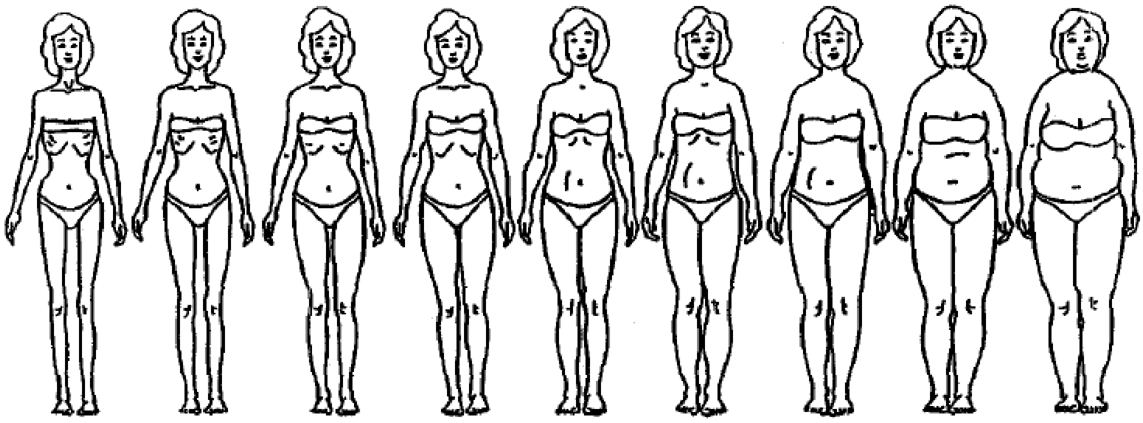

1 2 3 4 5 6 7 8 9

א. סמני את מספר הדמות שמייצגת את <u>דימוי הגוף הנוכחי שלך</u> - איך את רואה את עצמך כרגע

9 8 7 6 5 4 3 2 1

ב. סמני את מספר הדמות שמייצגת את <u>דימוי הגוף האידיאלי</u> עבורך - <u>איך שהיית רוצה ויכולה להיראות</u>:

9 8 7 6 5 4 3 2 1

6. הצלליות הבאות מייצגות דמויות שונות. אנא בחר מתוכן את הדמויות הרלבנטיות עבור ארבע השאלות הבאות:

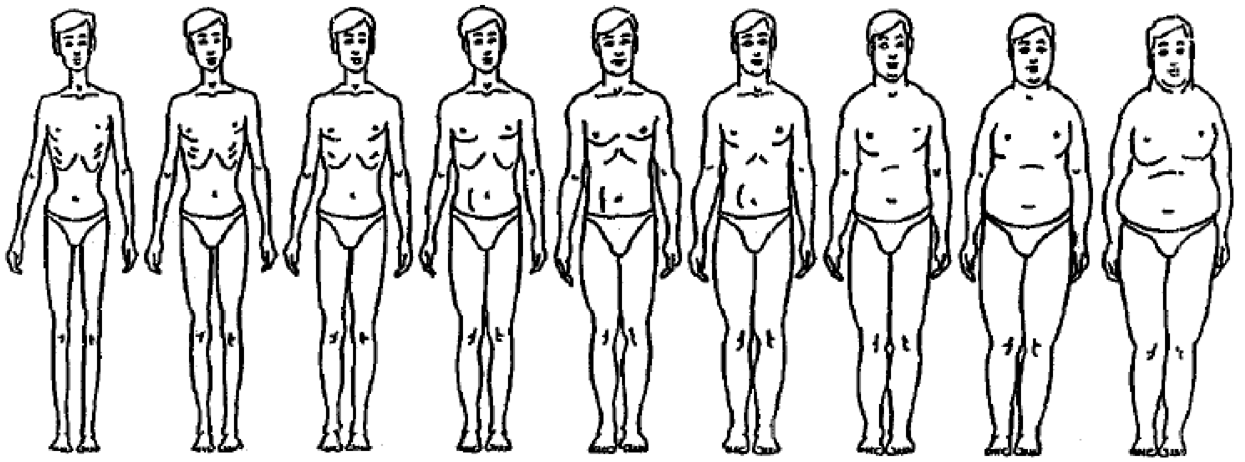

1 2 3 4 5 6 7 8 9

א. סמן את מספר הדמות שמייצגת את <u>דימוי הגוף הנוכחי שלך</u> - איך אתה רואה את עצמך כרגע

9 8 7 6 5 4 3 2 1

ב. סמן את מספר הדמות שמייצגת את דימוי הגוף האידיאלי עבורך - איך שהיית רוצה ויכול להיראות:

9 8 7 6 5 4 3 2 1

7. ביחס להתנהגות האכילה שלך:	אף פעם	לעיתים רחוקות	לפעמים	לעיתים קרובות	בדרך כלל	תמיד
1. אני אוכל/ת ממתקים ופחמימות ללא רגשי אשמה.	0	0	0	1	2	3
2. אני חושב/ת שה שהבטן גדולה מידי.	0	0	0	1	2	3
3. אני חושב/ת על דיאטה.	0	0	0	1	2	3
4. אני חושב/ת שהירכיים שלי רחבים מידי.	0	0	0	1	2	3
5. אני מרגיש/ה אשמה במיוחד לאחר אכילת יתר.	0	0	0	1	2	3
6. אני חושב/ת שהבטן שלי בגודל הנכון.	0	0	0	1	2	3
7. אני פוחד/ת מאוד מעליה במשקל.	0	0	0	1	2	3
8. אני מרוצה ממבנה גופי.	0	0	0	1	2	3
9. אני מגזים/ה לגבי החשיבות של משקל גופי.	0	0	0	1	2	3
10.אני אוהב/ת את מבנה הישבן שלי.	0	0	0	1	2	3
11.אני מאוד עסוק/ה במשאלה להיות רזה.	0	0	0	1	2	3
12.אני חושב/ת שהמותניים שלי רחבות.	0	0	0	1	2	3
13.אם אעלה קילו אחד, אני חושש/ת שאמשיך לעלות במשקל	0	0	0	1	2	3
14. אני חושב/ת שהירכיים שלי בגודל הנכון.	0	0	0	1	2	3
15. אני חושב/ת שהישבן שלי רחב מידי.	0	0	0	1	2	3

7. עד כמה קיימת אצלך הסכמה עם המשפטים הבאים	אף פעם	לעיתים רחוקות	לפעמים	לעיתים קרובות	תמיד
[1]. מוצא חן בעיני איך שאני נראה/נראית בתמונות	1	2	3	4	5
[2]. אנשים אחרים חושבים שאני ניראה/נראית טוב	1	2	3	4	5
[3]. אני גאה בגוף שלי	1	2	3	4	5
[4]. יש לי מחשבות טורדניות/חוזרות ביחס לצורך לשנות ולתקן את הגוף שלי	1	2	3	4	5
[5]. אני חושבת שהמראה שלי יעזור לי להשיג עבודה	1	2	3	4	5
[6].כשאני מסתכלת במראה אני אוהב/ת מה שניבט אלי	1	2	3	4	5
[7].יש הרבה מאוד דברים במראה שלי שהייתי משנה אם הייתי יכולה	1	2	3	4	5
[8]. אני מרוצה מהמשקל שלי	1	2	3	4	5
[9]. הלוואי והמראה שלי היה יותר טוב	1	2	3	4	5
[10]. מוצא חן בעיני כמה שאני שוקל/ת	1	2	3	4	5
[11]. הלוואי שהייתי נראית כמו מישהו אחר	1	2	3	4	5
[12]. אנשים בגילי אוהבים את המראה שלי	1	2	3	4	5
[13]. המראה שלי מדכדך אותי	1	2	3	4	5
[14]. אני נראית טוב כמו מרבית האנשים	1	2	3	4	5
[15]. אני די מרוצה מהמראה שלי	1	2	3	4	5
[16]. אני מרגישה שאני שוקלת נכון ביחס לגובה שלי	1	2	3	4	5
[17]. אני מרגישה בושה ביחס למראה שלי	1	2	3	4	5
[18]. כשאני שוקלת את עצמי זה מכניס אותי לדיכאון	1	2	3	4	5
[19]. המשקל שלי גורם לי להיות לא מאושרת	1	2	3	4	5

[20]. המראה שלי עוזר לי להיות פופולרית בקרב בני המין השני	1	2	3	4	5
[21]. אני מודאגת מאיך שאני ניראה/נראית	1	2	3	4	5
[22]. אני חושב/ שיש לי גוף טוב	1	2	3	4	5
[23]. אני נראית טוב כמו שאני רוצה	1	2	3	4	5

<u>שאלות שביעות רצון מהתכנית</u>

1. **מאילו מפגשים של התוכנית בעד עצמי היית?** מפגש מקדים 1 2 3 4 5 6 7 8
2. **מאילו מפגשים נעדרת?** מפגש מקדים 1 2 3 4 5 6 7 8
3. **עד כמה נושאי התוכנית היו לשביעות רצונך?** 1. מיעוטם 2. חלקם 3. רובם 4. כולם
4. **עד כמה הפעילויות באמצעותן הועבר הנושא עוררו עניין (במידה ופעילות מסוימת <u>לא הועברה דלגו</u> על השורה):**

פעילות היכרות ובניית החוזה הקבוצתי	כלל לא	מעטה	רבה	רבה מאוד
פעילות 1- העץ שלי- בניית הגזע, שורשים וקרקע	1	2	3	4
פעילות 2- המשך בניית עץ החיים- ענפים, עלים ופירות	1	2	3	4
פעילות 3- תדמית לעומת מהות- סטריאוטיפים	1	2	3	4
פעילות 4- השפעת התקשורת על העץ שלי - מה גורם לנו אושר?	1	2	3	4
פעילות 5- התמודדות עם קושי בגיל ההתבגרות עם חיזוק העצמאות ולקיחת אחריות והתמודדות עם תקשורת בין אישית שלי	1	2	3	4
פעילות 6- המראה שלי- דימוי גוף	1	2	3	4
פעילות 7- סערות החיים- התמודדות עם קושי	1	2	3	4
פעילות 8- חגיגת החיים- סיכום הפעילויות	1	2	3	4

5. דרג/י את שביעות הרצון הכללית שלך מהתוכנית 1. נמוכה מאוד 2.נמוכה 3. בינונית 4. גבוהה

6. ציינ/י את מספר הפעולה ושם הפעילות בה נתקלת בקושי רגשי שגרם לך אי נוחות או רצון שיחליפו את הפעילות?

____

7. מה היית רוצה שנוסיף לתוכנית?______________________________________

8. מה היית רוצה שנגרע מהתוכנית?______________________________________

תודה רבה!

____

בקשה לאישור ועדת אתיקה לביצוע מחקר בבני אדם[2]

תאריך: 23.6.2015

שם החוקר/ת: פרופ' מוריה גולן

שם המחקר: בעד עצמי –תכנית לקדום דמוי עצמי ודמוי גוף חיוביים בקרב מתבגרים בתחילת ובאמצע גיל ההתבגרות

I. המשתתפים:

1. **מספר הנבדקים/המשתתפים:**	400

2. **טווח גיל:** 10-15 קטינים (מתחת לגיל 18) – **נדרש טופס הסכמת הורים**/בוגרים

3. האוכלוסייה: ☐ סטודנטים ו/או בני משפחותיהם ☐ אוכלוסיה בגירה ונטולת חולשות

X אוכלוסיה אחרת (נא לפרט כגון: ילדי בי"ס, מורים, וכו')
ילדי בית ספר עממי וחטיבת ביניים

4. **הדרך להשגת השתתפותם** (הסבר מפורט):

פניה לבתי הספר להשתתפות במחקר קליני מבוקר עם חלוקה אקראית לקבוצת מחקר וביקורת – בדומה למה שנעשה על ידי קבוצת המחקר שלנו בשנים קודמות.

II. שיטות - סמן את __כל__ האפשרויות – __כן או לא__

		__כן__	__לא__
1	העברת שאלונים	X	
2	התערבות קלינית שבוחנת תכנית מניעה	X	

III. האם המכתב (טופס הסכמה מדעת) למשתתפים הפוטנציאליים כולל?

		__כן__	__לא__
1	תאור המחקר, שמו ומטרתו	X	
2	תועלת המחקר	X	
3	סיכויים ו/או סיכונים לנחקר	X	
4	המשימות המוטלות על הנחקר	X	
5	הצהרה על השתתפות רצונית וזכות המשתתפים לפרוש מהמחקר בכל עת ללא השלכות שליליות	X	
6	הבטחת סודיות, אנונימיות ופרטיות	X	
7	שם החוקר/ת וטלפון או דרך אחרת להתקשר עמו/ה	X	
8	טופס הסכמה להשתתפות רצונית	X	

		__כן__	__לא__
1	הולכת שולל או הסבר הלוקה בחסר		X
2	איסוף מידע רגיש		X

[2] מתוך הועדה להערכת מחקרים בבני אדם, אוניברסיטת חיפה.

3	חשיפה לגירויים היכולים להתקבל כמאיימים, מעליבים, מעוררי חרדה, מעוררי זכרונות טראומטיים או כדומה		X
4	חשיפה לגירויים פיזיים כגון: דרגות גבוהות של רעש או גירויים ויזואליים החורגים ממידת הגירוי היומיומי או כאב		X
5	איסוף מדדים ביולוגיים ו/או פיזיולוגיים (דם, רוק, דופק, לחץ דם, מדדים פיזיולוגיים אחרים).		X
6	שימוש בתרופות (נא לתאר את התרופות ואת האמצעים הננקטים לשמירה על בטחון המשתתפים)		X
7	מאמץ פיזי מעבר למקובל ביום-יום (נא לתאר את המשימה ואת האמצעים הננקטים להגנה על המשתתפים)		X
8	סיכון חברתי, משפטי, בראותי, כלכלי או אחר לנחקרים (כגון יצירת סטיגמה, סיכון סטטוס תעסוקתי או הפללה של נחקרים)		X
9	**פניה למשתתפים דרך גורם סמכותי** (מורים מטפלים מעבידים)	X	
10	**תגמול כספי, מתן ציון או אמצעים אחרים לעידוד ההשתתפות (נא לתאר)** הילדים יקבלו מתנה סמלית (בקבוק, כפכפים, צמיד סיליקון) בתמורה לזמן שהשקיעו במילוי כל אחד מהשאלונים	X	

III. הגנה על המשתתפים

	כן	לא
2. האם טופסי ההסכמה להשתתפות רצונית ישמרו בנפרד מתוצאות הנבדקים?	X	
2. האם תפורסם זהות המשתתפים?		X
3. **מלבד החוקר, האם יוכלו גורמים נוספים לעיין בתוצאותיהם של המשתתפים?** סטטיסטיקאי ותלמידות המוסמך המחקרי	X	

4. האם המחקר כולל סיכונים ו/או השלכות שליליות אפשריות על הנחקרים? אם כן, אילו אמצעים יינקטו על מנת להפחית את הסיכון לתוצאות כאלה
לא כולל סיכונים
5. במקרה של נזק לנחקר, אלו דרכי התערבות ינקטו? לא רלבנטי
6. נא לתאר את האמצעים אשר ינקטו להגן על זהות המשתתפים, והנתונים המתקבלים
לאחר שכל ילד מילא 3 שאלונים הם יחוברו לערכה אחת ויועברו על ידי עוזרת המחקר של התוכנית אל הסטטיסטיקאי. הסטטיסטיקאי יקבל קובץ משותף ללא שמות הילדים ויעביר את הממצאים של העבוד הסטטיסטי לתלמידת המחקר אליה הוא משתייך.

הנני מצהיר/ה בזאת שהמידע הנ"ל מלא נכון ומדויק והצעת המחקר תואמת את הסטנדרטים הבינלאומיים והאוניברסיטאיים לניהול מחקר אתי.

שם החוקר/ת: פרופ' מוריה גולן

חתימת החוקר/ת:

החלטת ועדת האתיקה (לשימוש הועדה בלבד)
תאריך הדיון: 13.8.15 אישור: 13.8.15

שם החוקר/ת: פרופ' מוריה גולן

שם המחקר: בעד עצמי –תכנית לקדום דמוי עצמי ודמוי גוף חיוביים בקרב מתבגרים בתחילת ובאמצע גיל ההתבגרות

חתימת ראש הועדה

Translation of Protocol Synopsis:

Intervention: School-based preventive program "In Favor of Myself", designed for adolescents aged 10-16 years. The program focuses on promoting positive self-image, self-esteem and body image.
"In Favor of Myself" was approved for delivery in North of Israel, by the Israeli ministry of education as part of the school based "life skills" collection.

Study Design: Class-randomized controlled trials

Population: Adolescents, ages 13-14 from northern Israel.

Inclusion Criteria: All children from the available school in the north, in grade seven and eight that they, themselves and their parents signed an informed consent and filled the study assessments at least twice.

The research process:

1. After approval of Tel Hai IRB, the school sent all parents the study informed consent as well as information sheet. All parents should signed the letter of consent.
2. Randomization of schools into control and intervention and the intervention classes into intervention 1 and intervention 2.
3. Each class filled the computerized qualtrics questionnaires at baseline, program completion (2 months) and 3 months after program completion in the presence of the school's teachers, consultants and the research student. Participants' phone numbers were used for gathering the questionnaires of the same participants.

The questionnaire include sociodemographic questions, Rosenberg self-esteem questionnaire, Internalization of thin body ideals, advertisements strategies, perceived pressure from media images, body esteem, body image, EDI_II body dissatisfaction and thin persuasion subscales.

4. Program will be delivered only to students in the intervention groups. Each group will be consisted of 15 participants. The teacher will be asked to stay in the class during sessions.

5. Sample size was determined by the statistician. We used the average score and the standard deviation in Rosenberg scale, reported for adolescents in the literature.

[illegible]
